# Inferring Personalized and Race-Specific Causal Effects of Genomic Aberrations on Gleason Scores: A Deep Latent Variable Model

**DOI:** 10.3389/fonc.2020.00272

**Published:** 2020-03-13

**Authors:** Zhong Chen, Andrea Edwards, Chindo Hicks, Kun Zhang

**Affiliations:** ^1^Department of Computer Science, Xavier University of Louisiana, New Orleans, LA, United States; ^2^Department of Genetics, LSU Health Sciences Center New Orleans, New Orleans, LA, United States; ^3^Bioinformatics Core of Xavier RCMI Center for Cancer Research, Xavier University of Louisiana, New Orleans, LA, United States

**Keywords:** deep latent variable model, causal effect inference, prostate cancer, racial disparity, genomic aberrations, Gleason scores

## Abstract

Extensive research has examined socioeconomic factors influencing prostate cancer (PCa) disparities. However, to what extent molecular and genetic mechanisms may also contribute to these inequalities still remains elusive. Although various *in vitro, in vivo*, and population studies have originated to address this issue, they are often very costly and time-consuming by nature. In this work, we attempt to explore this problem by a preliminary study, where a joint deep latent variable model (DLVM) is proposed to *in silico* quantify the personalized and race-specific effects that a genomic aberration may exert on the Gleason Score (GS) of each individual PCa patient. The core of the proposed model is a deep variational autoencoder (VAE) framework, which follows the causal structure of inference with proxies. Extensive experimental results on The Cancer Genome Atlas (TCGA) 270 European-American (EA) and 43 African-American (AA) PCa patients demonstrate that ERG fusions, somatic mutations in SPOP and ATM, and copy number alterations (CNAs) in ERG are the statistically significant genomic factors across all low-, intermediate-, and high-grade PCa that may explain the disparities between these two groups. Moreover, compared to a state-of-the-art deep inference method, our proposed method achieves much higher precision in causal effect inference in terms of the impact of a studied genomic aberration on GS. Further validation on an independent set and the assessment of the genomic-risk scores along with corresponding confidence intervals not only validate our results but also provide valuable insight to the observed racial disparity between these two groups regarding PCa metastasis. The pinpointed significant genomic factors may shed light on the molecular mechanism of cancer disparities in PCa and warrant further investigation.

## Introduction

Prostate cancer (PCa) is the most commonly diagnosed non-skin cancer and the second leading cause of cancer mortality in American men (1). In the US, an estimated 164,690 new cases and 29,430 deaths occurred in 2018 (1). Compared with European-American (EA) men, African American (AA) men experience a 60% higher incidence rate and a 2.4 times higher mortality rate of PCa (1). Moreover, AA men are diagnosed at an earlier age with higher Gleason Scores (GSs) and prostate-specific antigen (PSA) levels (2, 3) and are more likely to have aggressive diseases than men of other ethnic groups.

There are many factors that influence racial disparities in PCa, and a number of socioeconomic, cultural, and environmental factors have been identified (4–8). For example, unequal access to health care, diet, age, lifestyle, and family history strongly affect the race-specific PCa incidence and mortality rates. Other factors, such as poverty, lack of education, stigma, and type and aggressiveness of treatment have also been suggested as potential contributors to the disparity (9, 10). However, many studies have reported that the inequity remains even after those socioeconomic and treatment differences are adjusted (11). Ever-increasing evidence, on the other hand, suggests that a number of intrinsic molecular determinants specific to malignant cells, including genetic and/or genomic aberrations, must partially account for the observed health disparities (12, 13). For example, Edward et al. (14) reported that BRCA2 mutation is a potential risk factor associated with PCa incidences. Scott et al. (15) showed a much higher rate of cytochrome c oxidase subunit I (COI) mutation present in AA individuals, indicating its importance in racial disparity for PCa.

Compared to other cancer types, PCa is characterized by extraordinary genetic and genomic complexities (16, 17). Multiple studies have identified recurrent somatic mutations, copy number alterations (CNAs), and oncogenic structural DNA rearrangements in primary PCa (18–23). These include point mutations in SPOP, FOXA1, and TP53; CNAs involving MYC, RB1, PTEN, and CHD1; and ERG fusions, among other biologically relevant genes. Although certain gene mutations have been reported to be of importance in racial disparity for PCa (14, 15), the personalized and race-specific causal effects of other molecular aberrations on PCa aggressiveness (i.e., GS) have not yet been quantitatively realized, especially given large amounts of multiple clinical and high-throughput omics data.

To fill this gap, we propose a joint deep latent variable model, named DLVM, to *integratedly* estimate the personalized and race-specific causal effects that a genomic aberration may exert or not exert on a patient's GS. The core of DLVM is a deep variational autoencoder (VAE) framework, which follows the causal structure of inference with proxies. By deep learning multi-observational data of patients, DLVM is able to integrate the potential influence of immeasurable confounders or latent variables (e.g., interactions among interested genes) in its inferences. Exploratory studies on The Cancer Genome Atlas (TCGA) PCa tumor samples demonstrate that DLVM can pinpoint genomic aberrations that may be of significance for racial disparities in PCa. Moreover, compared to another state-of-the-art deep inference method, DLVM achieves much higher precision in causal inferences. Further validation on an independent set and the assessment of the estimated genomic-risk scores not only mutually validate the results but also elucidate the observed racial disparity between these two groups regarding PCa metastasis.

## Materials and Methods

### Data and Studied Genomic Aberrations

**Figure 1:**
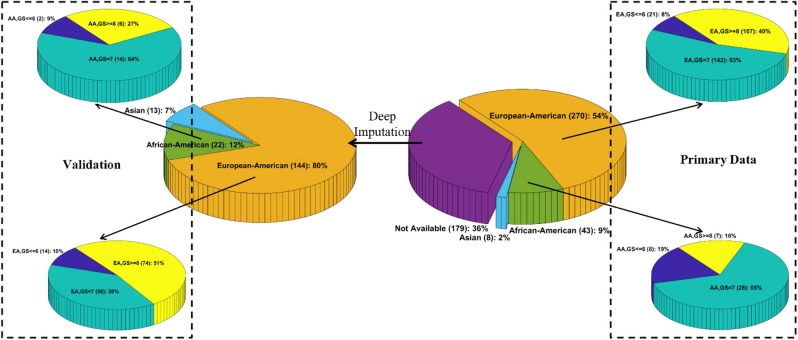
Patient distributions with respect to race and varied grades of Gleason scores in the primary and validation data, respectively.

We downloaded the multi-omics TCGA “Prostate Adenocarcinoma” (PRAD) dataset from cBioPortal^1^. The original dataset contains 270 EAs, 43 AAs, 8 Asians, and 179 patients whose race/ethnicity information is unavailable. To the best of our knowledge, the TCGA PRAD cohort is the only publicly available PCa dataset with the self-reported race/ethnicity information and matched multilevel genomic and clinical data. Those matched data, especially the clinical features, mutation profiles, and gene expression values, are essential for DLVM modeling. As such, we conducted the initial modeling on the primary set of 270 EA and 43 AA patients. Moreover, an independent validation was performed on the corresponding data of 166 patients (i.e., 144 EAs and 22 AAs) whose racial information is imputed by a published deep learning method (see *Race/Ethnicity Imputation*). The details of sources of data and the utilized features for model training are presented in Supplementary Text S1. Figure 1 illustrates the patient distributions with respect to varied grades of GS in the primary and validation datasets, where the high-risk/aggressive PCa is defined as GS ≥ 8, intermediate-risk PCa is defined as GS = 7, and low-risk/non-aggressive PCa is defined as GS ≤ 6 (24).

In this work, without further specifications, the studied genomic aberrations include ERG fusions; somatic mutations in SPOP, TP53, FOXA1, ATM, BRCA2, and PTEN; germline mutations in BRCA1 and BRCA2; and CNAs in LCP1, ERG, PTEN, and FOXA1. We choose these aberrations because they are the most frequent genomic alterations as summarized by cBioPortal^1^. Nevertheless, the potential values of these aberrations in PCa racial disparities are largely unknown. It is worth noting that when a specific genomic aberration is treated as the intervention variable, its gene expression values are excluded from the continuous variables to do the inferences.

### Race/Ethnicity Imputation

To obtain the unknown race or ethnicity of the 179 patients in the validation data, we utilized a deep imputation method similar to RIDDLE (25) to train a predictive model on the primary data with known racial information. Specifically, a multilayer perceptron (MLP) network that contains an input layer with 112 nodes (i.e., each node corresponds to a feature as described in Supplementary Text S1), two hidden layers with 512 Parametric Rectified Linear Unit (PReLU) nodes, and a *softmax* output layer with three nodes (i.e., each corresponds to EA, AA, and Asian) is designed to impute the racial information. Out of the 179 patients, 144, 22, and 13 patients are predicted to be EAs, AAs, and Asians, respectively (Figure 1).

### Method and Implementation of Deep Latent Variable Model

To learn the personalized causal effect of an event or intervention on a certain outcome, one has to know the true or factual outcomes with and without the intervention. However, in reality, one can only observe one of the outcomes and the other has to be reconstructed and inferred (Table 1). To infer the unknown outcome from observational data, confounders, i.e., the factors that affect the intervention, its outcomes and the observed noisy variables, need to be carefully handled. In our study, the intervention is an interested genomic aberration, the outcome is the GS of an individual AA or EA patient, and the observed noisy variables are the patients' multi-omics and clinical data. As PCa disparity is a multifactorial construct, it is reasonable to assume that multiple confounders, observed and latent, affect the way that a genomic aberration impacts a patient's GS. Specifically, an example of observed confounders is gene expression data, and examples of latent confounders include the crosstalk/interactions among genes or unseen influences between genotypes and clinical features. As a result, we propose a joint DLVM to tackle this problem.

**Table 1:** Different scenarios of causal effect inferences: Ideal-World vs. Real-World vs. Model-Inference [e.g., deep latent variable model (DLVM)].

**Patients**	**Ideal world**		**Real world**		**Reconstruction and inference (DLVM)**	
	Both true outcomes [a patient's Gleason Score (GS)] are known for a genomic aberration occurs (*t* = 1) and not occur (*t* = 0)		Only one true outcome (a patient's GS) is known for a genomic aberration occurs (*t* = 1) or not occur (*t* = 0)		Both outcomes (a patient's GS) can be estimated for a genomic aberration occurs (*t* = 1) and not occur (*t* = 0)	
1	*y*_1_ (0)	*y*_1_ (1)	*y*_1_ (0)	?	*ŷ*_1_ (0)	*ŷ*_1_ (1)
….	….	….	….	….	….	….
*n*	*y*_*n*_ (0)	*y*_*n*_ (1)	?	*y*_*n*_ (1)	*ŷ*_*n*_ (0)	*ŷ*_*n*_ (1)

**Figure 2:**
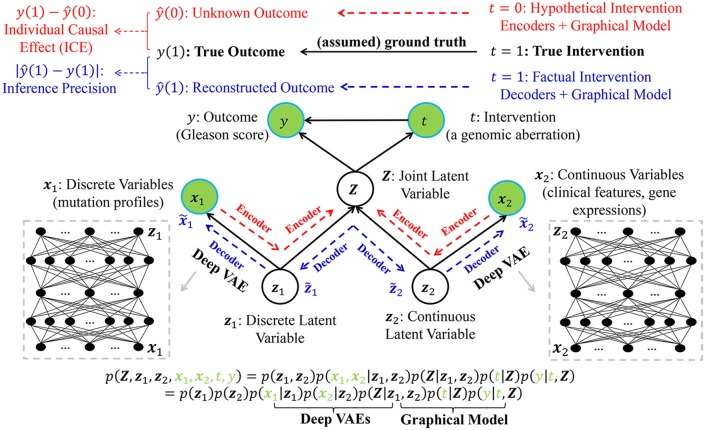
The graphical representation of the proposed deep latent variable model (DLVM) with two processes for reconstruction of the true outcome (in blue) and estimation of the unknown outcome (in red), respectively. Please see detailed descriptions in *Method and Implementation of Deep Latent Variable Model*.

Without loss of generality, we assume that the true intervention is *t* = 1 and the true outcome is *y*(1) for a certain patient (e.g., the *n*-th patient in Table 1). Figure 2 demonstrates the underlying inference mechanism of DLVM. In DLVM, *y* denotes the outcome (e.g., GS of a patient); *t* represents the intervention (e.g., a specific genomic aberration); **x**_1_ and **x**_2_, respectively, denote the observed discrete and continuous noisy multi-omics and clinical variables; **z**_1_ and **z**_2_, respectively, denote the discrete and continuous latent confounders, and **Z** represents the joint latent confounder. We assume that the joint distribution *p*(**Z**, **z**_1_, **z**_2_, **x**_1_, **x**_2_, *t, y*) of the latent confounders and the observed data can be approximately reconstructed from the observations (**x**_1_, **x**_2_, *t, y*). As indicated by blue and red, respectively (Figure 2), there are two processes in DLVM: reconstruction of the true outcome [i.e., *ŷ*(1)] and estimation of the unknown outcome [i.e., *ŷ*(0)]. More specifically, in both processes, we first parameterize the discrete prior distribution *p*(**z**_1_) as a Bernoulli distribution and the continuous prior distribution *p*(**z**_2_) as a Gaussian distribution. Then, the conditional distributions *p*(**x**_1_|**z**_1_), *p*(**x**_2_|**z**_2_), and *p*(**Z**|**z**_1_, **z**_2_) can be obtained via deep decoders or encoders of the VAEs (27). After that, we use the graphical representation to compute *p*(**Z, ***t, y*) = *p*(**Z**)*p*(*t*|**Z**)*p*(*y*|*t*, **Z**), whereby the overall joint distribution can be estimated by *p*(**Z**, **z**_1_, **z**_2_, **x**_1_, **x**_2_, *t, y*) = *p*(**z**_1_)*p*(**z**_2_)*p*(**x**_1_|**z**_1_)*p*(**x**_2_|**z**_2_)*p*(**Z**|**z**_1_, **z**_2_)*p*(*t*|**Z**)*p*(*y*|*t*, **Z**)(Please refer to the detailed discussion in Supplementary Text S2). Finally, given the assumed true intervention *t* = 1, reconstruction of the true outcome [i.e., *ŷ*(1)] and estimation of the unknown outcome [i.e., *ŷ*(0)] can be achieved by using the estimated joint distribution *p*(**Z**, **z**_1_, **z**_2_, **x**_1_, **x**_2_, *t, y*). In this way, for the outcome of a certain patient (i.e., GS in our case), the reconstruction precision and individual causal effect (ICE) can be computed by |*ŷ*(1) − *y*(1)| and *y*(1) − *ŷ*(0), respectively.

In summary, using VAEs as its core inference technique, DLVM is able to derive the complex non-linear relationships between (**x**_1_, **x**_2_) and (**Z**, **z**_1_, **z**_2_, *t, y*) and approximately reconstruct *p*(**Z**, **z**_1_, **z**_2_, **x**_1_, **x**_2_, *t, y*). This capability allows DLVM to further infer the unknown outcome given an counterfactual/hypothetical intervention (e.g., *t* = 0) (via the deep encoder) and approximate the true outcome given a truthful/factual intervention (e.g., *t* = 1) (by the deep decoder). The details of DLVM modeling, inference, and optimization (with theoretical proofs) are presented in Supplementary Text S2. From a technical perspective, DLVM is a variant of causal effect inference methods with latent variable modeling capability. The interested readers can refer to Kingma and Welling (26), Box and Tiao (27), Goodfellow et al. (28), Lee et al. (29), Louizos et al. (30), and Yoon et al. (31) for reviews of related work.

In this study, we prototype DLVM by two deep VAEs with three hidden layers for discrete and continuous latent variables. Each of the first two hidden layers contains 300 neurons, and there are 100 neurons in the third one. We adopt the sigmoid activation function in each hidden layer and the *softmax* function in the output layer. The dimensions of the latent confounders **Z**, **z**_1_ and **z**_2_ are set as 50, 160, and 160, respectively. The DNN is trained using the gradient descent algorithm with up to 300 epochs, a batch size of 10, a learning rate of 1e−5, and a decay rate of 1e−5. ADAM (32) is used as the optimizer, and all model parameters are determined via a preliminary validation process. DLVM is implemented by modifying the source code of CEVAE^2^ with the utilization of additional Python packages, such as numpy, sklearn, tensorflow, and PyTorch.

### Genomic-Risk Scores Obtained via Deep Latent Variable Model Estimation

As DLVM is able to estimate the GS for each patient assuming that an interested genomic aberration does occur (i.e., *t* = 1), we also compute the genomic risk scores (GRSs) based on the DLVM's estimates so that the impacts of these genomic aberrations can also be interpreted at the population level regarding PCa metastasis. Specifically, based on the obtained GSs from DLVM, a genomic risk assessment model as documented in Mahal et al. (24) and Spratt et al. (33) is first used to compute the GRSs, and then, we compare these aberration-specific GRSs between AAs and EAs by a two-sided (α = 0.05) statistical *t*-test. The implementations are performed with R 3.6.0 (R package: EBPRS). It is worth noting that the output GRSs are continuous between 0 and 1, with higher scores indicating a greater risk of PCa metastasis.

### Measurement Metrics and Experimental Design

To quantitatively assess to what extent a studied genomic aberration may affect the GS of each individual patient, we train DLVM with the GS as the outcome (i.e., *y*) and each genomic aberration as a binary intervention variable (i.e., *t* = 1 *or* 0). Let *y*_*i*_(0) and *y*_*i*_(1) be the true outcomes of the *i*th patient when *t* = 0 and *t* = 1 and *ŷ*_*i*_(0) and *ŷ*_*i*_(1) be the outcomes estimated by DLVM, the individual causal effect (ICE) for the *i*-th patient can be measured by *ŷ*_*i*_(1) − *y*_*i*_(0) (if the studied aberration does not occur, i.e., *t* = 0) or *y*_*i*_(1) − *ŷ*_*i*_(0) (if the studied aberration occurs, i.e., *t* = 1). The population causal effect for a racial group can be estimated by the average ICE (AICE), which is defined as: $AICE=\sqrt{\frac{\underset{i\in \left\{i|t=0\right\}}{\sum }{\left({\hat{y}}_{i}\left(1\right)-y_{i}\left(0\right)\right)}^{2}+\underset{i\in \left\{i|t=1\right\}}{\sum }{\left(y_{i}\left(1\right)-\;{\hat{y}}_{i}\left(0\right)\right)}^{2}}{n}}$, where *n* is the number of samples in a group. We use root mean square error (RMSE) defined as follows to measure the precision of the causal inference process: $RMSE=\sqrt{\frac{\underset{i\in \left\{i|t=0\right\}}{\sum }{\left({\hat{y}}_{i}\left(0\right)-y_{i}\left(0\right)\right)}^{2}+\underset{i\in \left\{i|t=1\right\}}{\sum }{\left({\hat{y}}_{i}\left(1\right)-y_{i}\left(1\right)\;\right)}^{2}}{n}}$.

In the experiments, we report the average AICE and RMSE over 10 replications of 3-fold cross validations on each racial group. In each fold, ~47, 20, and 33% of the samples in a racial group are used for model training, validation, and testing purposes. This experimental design ensures that all the samples are used for model performance assessment, i.e., calculations of AICE and RMSE.

## Experimental Results

### Inference Precision Comparison to a Benchmark Method on the Primary Data

**Figure 3:**
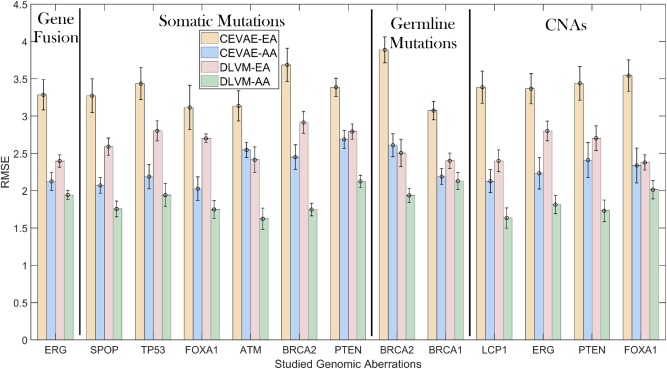
Inference precision comparisons measured by root mean square errors (RMSEs): deep latent variable model (DLVM) vs. causal effect variational autoencoder (CEVAE) on African Americans (AAs) and European-Americans (EAs) in the primary data. CNAs, copy number alterations.

To demonstrate the inference precision of DLVM, we first compare it with one of the state-of-the-art inference methods [i.e., causal effect VAE (CEVAE) (30)] w.r.t. the RMSE metric using the primary data. As shown by Figure 3 and Supplementary Table S1, for each racial group, DLVM consistently achieves lower RMSEs than CEVAE in causal effect inferences over all studied genomic aberrations with *p* < 0.0001. Compared to CEVAE, the overall average reductions in inference RMSEs achieved by DLVM on AAs and EAs are 19.03 and 22.98%, respectively. Specifically, in terms of ERG fusions, the average RMSEs of DLVM on AAs and EAs are 8.54 and 27.02% lower than those of CEVAE. With respect to the studied somatic mutations, the average RMSEs of DLVM on AAs and EAs are 21.03 and 19.01% lower than those of CEVAE. As to the germline mutations (or CNAs), the average RMSEs of DLVM on AAs and EAs are 14.26 and 28.70% (or 21.03 and 25.06%) lower than those of CEVAE. These results indicate that, compared to CEVAE, DLVM is more precise and reliable in inferring the race-specific causal effects that a genomic aberration may pose on GS.

### Identifying Race-Specific Genomic Aberrations via Average Individual Causal Effects on the Primary Data

To determine potential genomic aberrations of significance for racial disparities in PCa aggressiveness, we compare the race-specific AICEs of the studied genomic aberrations for low-grade (GS ≤ 6), intermediate-grade (GS = 7), and high-grade (GS ≥ 8) EA and AA patients on the primary data. It is worth noting that AICE can assess the population causal effect of a genomic aberration with respect to different GSs. In addition to AICE, DLVM is able to estimate the ICE for each patient (see Materials and Methods).

**Figure 4:**
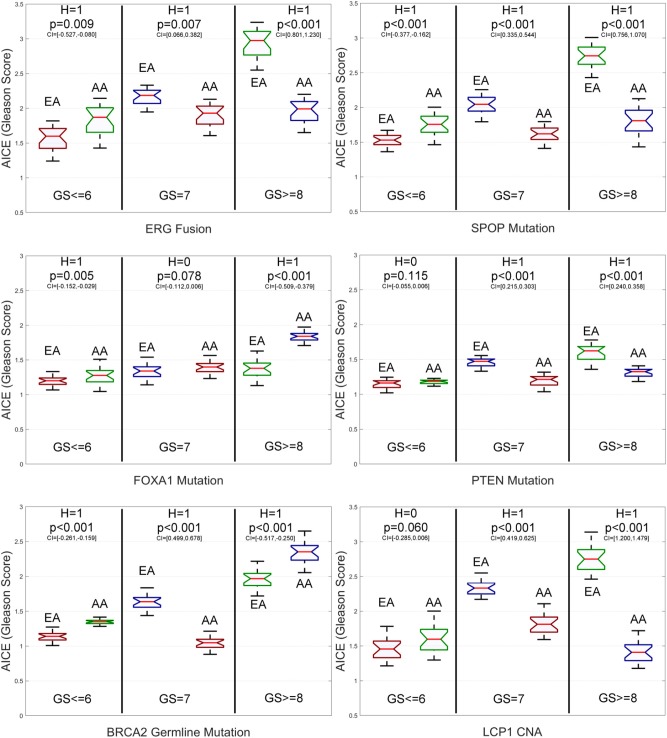
Box plots of six genomic-aberration specific average individual causal effects (AICEs) of African Americans (AAs) and European-Americans (EAs) in the primary data for different grades of Gleason Score (GS). The paired *t*-test with the significance level α = 0.05 is utilized for hypothesis testing, where the null hypothesis is “*H* = 0: genomic aberration-specific AICEs are not differentiated over racial groups” and the alternative hypothesis is “*H* = 1: genomic aberration-specific AICEs are differentiated over racial groups.” CI, confidence interval.

From Figure 4 and Supplementary Figure S1, we have the following observations. First, for low-grade patients, AICEs of AAs are statistically significantly higher than those of EAs over most of the studied genomic aberrations, including ERG fusions, somatic mutations in SPOP, FOXA1 and BRCA2, BRCA2 and BRCA1 germline mutations, and CNAs in ERG and FOXA1. The AICE of EAs is statistically significantly higher than that of AAs only on somatic mutations in ATM. There is no statistically significant difference in AICEs of AAs and EAs for TP53 and PTEN mutations as well as LCP1 and PTEN CNAs. Second, regarding intermediate-grade patients, AICEs of EAs are statistically significantly higher than those of AAs on ERG fusions, somatic mutations in SPOP, PTEN, ATM and TP53, BRCA2 germline mutation, and CNAs in LCP1, ERG, and PTEN. There is no statistically significant difference in AICEs of AAs and EAs for FOXA1 and BRCA2 mutations, BRCA1 germline mutation, and FOXA1 CNAs. Third, for high-grade patients, AICEs of AAs are statistically significantly higher than those of EAs on BRCA2 germline mutation, BRCA2 and FOXA1 somatic mutations, and CNAs in FOXA1; while AICEs of EAs are statistically significantly higher than those of AAs on ERG fusions, somatic mutations in SPOP, PTEN, ATM, and TP53 and CNAs in ERG and LCP1. There is no statistically significant difference in AICEs of AAs and EAs for BRCA1 germline mutation and CNAs in PTEN. The results for each grade of PCa patients suggest that: (1) Those identified significant genomic aberrations could be important molecular determinants of the racial disparity in the corresponding grade of tumors; and (2) For patients within the same grade category, each of those aberrations may pose a larger impact on AAs (or EAs) than EAs (or AAs) depending on which racial group of a higher AICE. Lastly, AICEs are statistically significantly differentiated between AAs and EAs across all three grades of GS with respect to ERG fusions, somatic mutations in SPOP and ATM, BRCA2 germline mutation, and CNAs in ERG compared to other genomic aberrations. The details of all estimated race-specific AICEs and related statistics for different grades of GS are summarized in Supplementary Table S2.

### Genomic Risk Score-Based Assessment on the Primary Data

**Figure 5:**
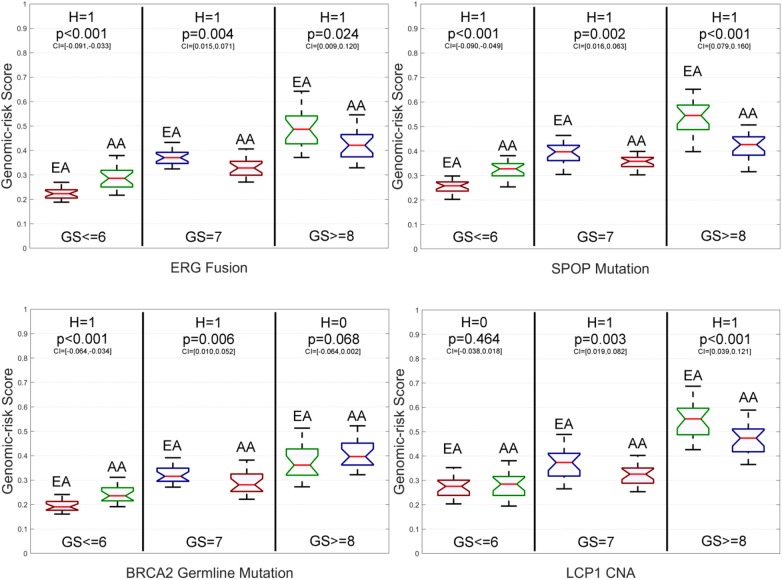
Box plots of six genomic aberration-specific genomic risk scores (GRSs) of African Americans (AAs) and European Americans (EAs) in the primary data for different grades of Gleason Score (GS). The paired *t*-test with the significance level α = 0.05 is utilized for hypothesis testing, where the null hypothesis is “*H* = 0: genomic aberration-specific GRSs are not differentiated over racial groups” and the alternative hypothesis is “*H* = 1: genomic aberration-specific GRSs are differentiated over racial groups.” CI, confidence interval.

To further explore the impacts of the studied genomic aberrations at the population level regarding PCa metastasis, we report the genomic aberration-specific GRSs of EAs and AAs in the primary data w.r.t. different grades of GS in Figure 5, Supplementary Figure S2, and Supplementary Table S3. In fact, as shown by Supplementary Table S4, the race-specific GRS patterns that the genomic aberrations impact patients' GSs are quite similar to the race-specific patterns observed for AICEs. Please see the implications of such a similarity in the *Discussion* section.

Specifically, for low-grade patients, we also find that: (1) GRSs of AAs are statistically significantly higher than those of EAs on ERG fusions, somatic mutations in SPOP, FOXA1 and BRCA2, BRCA2 and BRCA1 germline mutations, and CNAs in ERG and FOXA1; (2) The GRS of EAs is statistically significantly higher than that of AAs only on somatic mutations in ATM; and (3) There is no statistically significant difference in GRSs of AAs and EAs for TP53 and PTEN mutations as well as LCP1 and PTEN CNAs. As to intermediate-grade patients, GRSs of EAs are also statistically significantly higher than those of AAs on ERG fusions, somatic mutations in SPOP, PTEN, ATM, and TP53, BRCA2 germline mutations, and CNAs in LCP1, ERG, and PTEN. There is no statistically significant difference in GRSs of AAs and EAs for BRCA2 mutations, BRCA1 germline mutations, and FOXA1 CNAs. Compared to the AICE patterns of this grade category, one difference is that the GRS of AAs is statistically significantly higher than that of EAs on somatic mutations in FOXA1. For high-grade patients, GRSs of AAs are statistically significantly higher than those of EAs on BRCA2 and FOXA1 somatic mutations and CNAs in FOXA1, while GRSs of EAs are statistically significantly higher than those of AAs on EGR fusions, somatic mutations in SPOP, PTEN, ATM, and TP53, and CNAs in ERG and LCP1. There is no statistically significant difference in GRSs of AAs and EAs for BRCA1 and BRCA2 germline mutations and CNAs in PTEN. Across all three grades of GS, GRSs are statistically significantly differentiated between AAs and EAs with respect to ERG fusions, somatic mutations in SPOP, FOXA1, and ATM, and CNAs in ERG compared to other genomic aberrations.

### Validation of the Identified Race-Specific Genomic Aberrations

We further test our proposed method on the validation data containing 144 EAs and 22 AAs, where patients' racial information is inferred using the deep imputation method (25). Similarly, we compare the race-specific AICEs of the studied genomic aberrations for low-grade (GS ≤ 6), intermediate-grade (GS = 7), and high-grade (GS ≥ 8) EA and AA patients.

**Figure 6:**
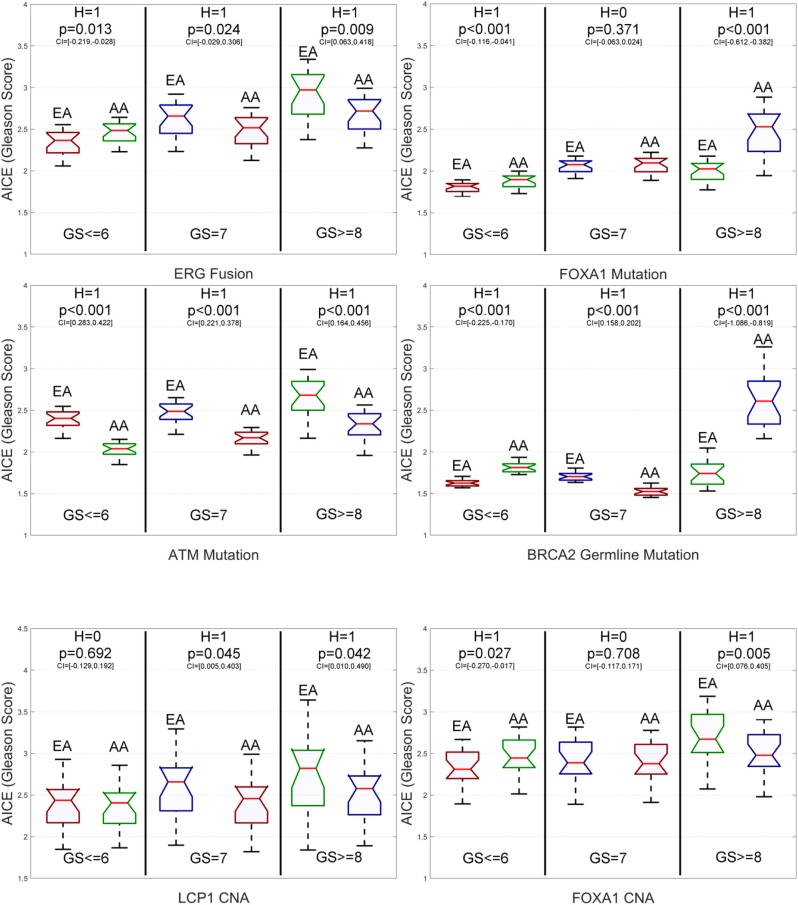
Box plots of six genomic aberration-specific average individual causal effects (AICEs) of African Americans (AAs) and European Americans (EAs) in the validation data for different grades of Gleason Score (GS). The paired *t*-test with the significance level α = 0.05 is utilized for hypothesis testing, where the null hypothesis is “*H* = 0: genomic aberration-specific AICEs are not differentiated over racial groups” and the alternative hypothesis is “*H* = 1: genomic aberration-specific AICEs are differentiated over racial groups.” CI, confidence interval.

From Figure 6 and Supplementary Figure S3, we have the following observations. First, for low-grade patients, AICEs of AAs are statistically significantly higher than those of EAs on ERG fusions, somatic mutations in SPOP, FOXA1 and BRCA2, BRCA2 and BRCA1 germline mutations, and CNAs in ERG and FOXA1. AICEs of EAs are statistically significantly higher than those of AAs only on somatic mutations in ATM. There is no statistically significant difference in AICEs of AAs and EAs for TP53 and PTEN somatic mutations as well as LCP1 and PTEN CNAs. Second, regarding intermediate-grade patients, AICEs of EAs are statistically significantly higher than those of AAs on somatic mutations in TP53, ATM, and PTEN, BRCA2 germline mutation, and CNAs in LCP1, ERG, and PTEN. There is no statistically significant difference in AICEs of AAs and EAs for nearly one third of the studied genomic aberrations, i.e., somatic mutations in SPOP and FOXA1, BRCA1 germline mutations, and CNAs in FOXA1. Lastly, for high-grade patients, AICEs of EAs are statistically significantly higher than those of AAs on ERG fusions, SPOP, TP53, ATM, and PTEN somatic mutations, and CNAs in LCP1, ERG, and FOXA1, while AICEs of AAs are statistically significantly higher than those of EAs on somatic mutations in FOXA1 and BRCA2 and BRCA2 germline mutation. There is no statistically significant difference in AICEs of AAs and EAs for BRCA1 germline mutation and CNAs in PTEN. The details of all estimated race-specific AICEs and related statistics for different grades of GS using the validation data are summarized in Supplementary Table S5.

In addition, as shown by Supplementary Table S6, the race-specific AICE patterns obtained on the primary data are quite similar to those obtained on the validation data. Among 39 comparisons with respect to 13 genomic aberrations and three grades, there are only three differences. They are somatic mutations in SPOP and BRCA2 for intermediate-grade patients and CNAs in FOXA1 for high-grade patients. Over 92% consistency rate between race-specific AICE patterns obtained on the primary and independent validation data further demonstrates the efficacy and robust capability of DLVM in causal inference.

## Discussion

Remarkable racial disparities have been reported in PCa incidence and mortality rates. In spite of these recognitions, precise mechanisms underlying these prevailing racial disparities remain poorly understood. Although socioeconomic factors play critical roles in such health disparities, increasing efforts have begun to explore molecular mechanisms in tumor biology and ancestry-related aspects that may be attributed to the observed PCa health disparities. However, most of these efforts are confined to expensive and time-consuming *in vitro, in vivo*, and population studies, where non-omics features and high-throughput multi-omics data cannot be well-integrated to infer potential race-specific causal biological determinants in an efficient and cost-effective manner.

To our knowledge, this is the first study to apply the machine learning approach (i.e., deep learning) to integratedly examine the personalized and race-specific causal effects that molecular aberrations may pose on classic PCa phenotypic risk factors (i.e., GS) via multi-omics and non-omics data. For 13 well-known molecular aberrations in PCa biology, we computationally explore their potentials as biological determinants of racial disparities in PCa via a novel DLVM and multiple population-level evaluation metrics (i.e., RMSEs, AICEs, and GRSs). By scrutinizing the TCGA PCa patients in different grades of GS and over all grades, we report race-specific AICEs, GRSs, related statistics, as well as the studied genomic aberrations that could contribute to PCa racial disparities. Some of the findings are consistent with what has been reported in the literature, which validates the results to a certain extent. For example, we find that for low-grade PCa, both AICEs and GRSs of AAs are statistically significantly higher than those of EAs over most of the studied genomic aberrations (i.e., ERG fusions, somatic mutations in SPOP, BRCA2, and FOXA1, germline mutations in BRCA2 and BRCA1, and CNAs in ERG and FOXA1). This indicates that AAs with low-grade PCa may suffer from higher tumor burdens and risks. As such, special care in addition to active surveillance should be given to AA men as they are more likely to die from low-grade PCa (34). This perception is consistent with the latest recommendations made by Mahal et al. (25, 35), National Cancer Institute (34), and Tsivian et al. (36).

Moreover, it is worth noting that the race-specific GRS patterns that the genomic aberrations affect patients' GSs highly resemble the race-specific patterns observed for AICEs. There are only two differences when these two sets of patterns are compared with respect to different grades of GS. One is that for intermediate-grade patients, AAs' GRS is statistically significantly higher than EAs' GRS on somatic mutations in FOXA1, while there is no statistically significant difference in AICEs of the two groups for the same aberration. The other is that for high-grade patients, AAs' AICE is statistically significantly higher than EAs' AICE on BRCA2 germline mutations, while there is no statistically significant difference in GRSs of the two groups for this alternation. Such a high similarity between these two result sets not only provides mutual verification but also indicates that the genomic aberrations directly identified as race-specific causal factors for PCa aggressiveness may also offer insights to PCa metastasis. We further compare the results obtained from AICEs and GRSs across patients in all three grades. We find that four shared genomic aberrations, ERG fusions, somatic mutations in SPOP and ATM, and CNAs in ERG, are the statistically significant genomic factors that may contribute to the disparities between these two groups. Some of these findings are largely consistent to the ethnic differences observed for *ERG* gene fusions and *SPOP* mutation (37, 38).

Compared to previous research, our study also leads to some new findings. For example, we find that: (1) for intermediate- and high-grade PCa, most of the studied aberrations (i.e., ERG fusions, somatic mutations in SPOP, TP53, ATM, and PTEN, CNAs in LCP1 and ERG) affect EAs more than AAs and (2) somatic mutations in ATM consistently impact EAs more over all three grades. In contrast to prior studies (11, 39), we do observe that somatic mutations in TP53 impacts intermediate- and high-grade patients, which is especially true for EAs. Part of this observation is consistent to the reported critical role of TP53 mutations in PCa tumor progression and metastasis (40, 41), which suggests that this gene might serve as a marker of disease aggressiveness and progression for PCa. All of these findings depict a small part of a complex picture of causal relations between race and tumor burden across the spectrum of PCa. Further efforts are warranted to validate the results and to enhance the capacity of the proposed deep model.

Due to some privacy and socioeconomic concerns of AA patients, most of the PCa data in the public domain belong to the European ancestry. To the best of our knowledge, the TCGA PRAD cohort is the only publicly available PCa dataset that contains a decent number of AA and EA patients with self-reported race/ethnicity as well as the matched multilevel genomic and clinical data. Those clinical and multi-omics data, especially the mutation profiles and gene expression values, are necessities for DLVM modeling. As a result, we carried out the model training on the primary data of 270 EA and 43 AA patients. Furthermore, an independent validation was performed on the corresponding data of 166 patients (including 144 EAs and 22 AAs), whose race/ethnicity was predicted by a deep imputation method (25). Over 92% consistency rate between the race-specific AICE patterns obtained on the primary and independent validation sets further demonstrates DLVM's ability in unbiasedly inferring the true causal effects between GS and an interested genomic aberration. As part of future work, we will impute PSA levels missing in the TCGA cohort using established methods so that our study can be extended to this widely used component of nearly all risk stratification methods for PCa. The co-effects of multiple alternations as the intervention will be further explored by coupling DLVM with some robust frequent pattern mining techniques (42, 43). Lastly, we will refine the proposed algorithm to take into account other variables, such as germline single-nucleotide polymorphisms (SNPs) and critical race-relevant socioeconomic and/or environmental factors in the modeling process for population-focused cancer research.

## Conclusion

In this paper, we introduce the first deep learning-based approach (i.e., DLVM) to inferring the personalized and race-specific causal effects that genomic aberrations (i.e., gene fusions, somatic mutations, germline mutations, and CNAs) may exert on PCa aggressiveness (i.e., GS). As PCa disparity is a multifactorial construct, DLVM assumes that multiple confounders, observed and latent, influence the way that genomic aberrations affect a patient's GS. As such, by jointly learning multi-observational data of patients, DLVM is able to incorporate the potential influence of immeasurable confounders or latent variables (e.g., interactions among interested genes) in its inferences. Thorough empirical studies and multiple evaluation metrics on 313 TCGA primary PCa and 166 samples (i.e., 144 EAs and 22 AAs) in an independent validation set demonstrate the efficacies of DLVM in identifying significant genomic factors that may contribute to the molecular basis of PCa racial disparities. Findings obtained through this study, once further validated, will shed light on developing molecular markers of predictive and prognostic values and therapeutic targets for men of African or European descent.

## Data Availability Statement

Publicly available datasets were analyzed in this study. This data can be found here: CBioPortal, Prostate Adenocarcinoma (TCGA, Cell 2015).

## Author Contributions

ZC and KZ designed the experiments with help from AE and CH. ZC performed the experiments. ZC and KZ analyzed the data. KZ, ZC, AE, and CH wrote the paper. KZ conceived and supervised this study. All the authors read and approved the final manuscript.

### Conflict of Interest

The authors declare that the research was conducted in the absence of any commercial or financial relationships that could be construed as a potential conflict of interest.

## Footnotes

^1^Available online at: https://www.cbioportal.org/datasets

^2^Available online at: https://github.com/rik-helwegen/CEVAE_pytorch

## References

[1] Siegel RL Miller KD Jemal A Cancer statistics, 2018. CA Cancer J Clin. (2018) 68:7–30. 10.3322/caac.21442 29313949

[2] Moul JW Sesterhenn IA Connelly RR Douglas T Srivastava S Mostofi FK. Prostate-specific antigen values at the time of prostate cancer diagnosis in African-American men. JAMA. (1995) 274:1277–81. 10.1001/jama.1995.03530160029029 7563532

[3] Powell IJ Banerjee M Novallo M Sakr W Grignon D Wood DP Prostate cancer biochemical recurrence stage for stage is more frequent among African-American than white men with locally advanced but not organ-confined disease. Urology. (2000) 55:246–51. 10.1016/S0090-4295(99)00436-7 10688088

[4] Du XL Fang S Coker AL Sanderson M Aragaki C Cormier JN. Racial disparity and socioeconomic status in association with survival in older men with local/regional stage prostate carcinoma: findings from a large community-based cohort. Cancer. (2006) 106:1276–85. 10.1002/cncr.21732 16475208

[5] Mahal BA Ziehr DR Aizer AA Hyatt AS Sammon JD Schmid M. Getting back to equal: the influence of insurance status on racial disparities in the treatment of African American men with high-risk prostate cancer. Urol Oncol. (2014) 32:1285–91. 10.1016/j.urolonc.2014.04.014 24846344

[6] Ziehr DR Mahal BA Aizer AA Hyatt AS Beard CJ Choueiri TK. Income inequality and treatment of African American men with high-risk prostate cancer. Urol Oncol. (2015) 33:18.e7–13. 10.1016/j.urolonc.2014.09.005 25306287

[7] Wu S Zhu W Thompson P Hannun YA. Evaluating intrinsic and non-intrinsic cancer risk factors. Nat Commun. (2018) 9:1–12. 10.1038/s41467-018-05467-z 30154431 PMC6113228

[8] Borno H George DJ Schnipper LE Cavalli F Cerny T Gillessen S. All men are created equal: addressing disparities in prostate cancer care. Am Soc Clin Oncol Educ Book. (2019) 39:302–8. 10.1200/EDBK_238879 31099647

[9] Powell IJ. Epidemiology and pathophysiology of prostate cancer in African-American men. J Urol. (2007) 177:444–9. 10.1016/j.juro.2006.09.024 17222606

[10] National Academies of Sciences, Engineering, and Medicine Communities in Action: Pathways to Health Equity. Washington, DC: National Academies Press (2017). 28418632

[11] Bhardwaj A Srivastava SK Khan MA Prajapati VK Singh S Carter JE. Racial disparities in prostate cancer: a molecular perspective. Front Biosci. (2017) 22:772–82. 10.2741/4515 27814645 PMC5242333

[12] Farrell J Petrovics G McLeod DG Srivastava S. Genetic and molecular differences in prostate carcinogenesis between African American and Caucasian American men. Int J Mol Sci. (2013) 14:15510–31. 10.3390/ijms140815510 23892597 PMC3759870

[13] Chornokur G Dalton K Borysova ME Kumar NB. Disparities at presentation, diagnosis, treatment, and survival in African American men, affected by prostate cancer. Prostate. (2011) 71:985–97. 10.1002/pros.21314 21541975 PMC3083484

[14] Edwards SM Kote-Jarai Z Meitz J Hamoudi R Hope Q Osin P. Two percent of men with early-onset prostate cancer harbor germline mutations in the BRCA2 gene. Am J Hum Genet. (2003) 72:1–12. 10.1086/345310 12474142 PMC420008

[15] Scott TA Arnold R Petros JA. Mitochondrial cytochrome c oxidase subunit 1 sequence variation in prostate cancer. Scientifica. (2012) 2012:701810. 10.6064/2012/701810 24124627 PMC3795349

[16] Barbieri CE Demichelis F Rubin MA. Molecular genetics of prostate cancer: emerging appreciation of genetic complexity. Histopathology. (2012) 60:187–98. 10.1111/j.1365-2559.2011.04041.x 22212086

[17] Beltran H Yelensky R Frampton GM Park K Downing SR MacDonald TY. Targeted next-generation sequencing of advanced prostate cancer identifies potential therapeutic targets and disease heterogeneity. Eur Urol. (2013) 63:920–6. 10.1016/j.eururo.2012.08.053 22981675 PMC3615043

[18] Baca SC Prandi D Lawrence MS Mosquera JM Romanel A Drier Y. Punctuated evolution of prostate cancer genomes. Cell. (2013) 153:666–77. 10.1016/j.cell.2013.03.021 23622249 PMC3690918

[19] Barbieri CE Baca SC Lawrence MS Demichelis F Blattner M Theurillat JP. Exome sequencing identifies recurrent SPOP, FOXA1 and MED12 mutations in prostate cancer. Nat Genet. (2012) 44:685. 10.1038/ng.2279 22610119 PMC3673022

[20] Berger MF Lawrence MS Demichelis F Drier Y Cibulskis K Sivachenko AY. The genomic complexity of primary human prostate cancer. Nature. (2011) 470:214–20. 10.1038/nature09744 21307934 PMC3075885

[21] Cooper CS Eeles R Wedge DC Van Loo P Gundem G Alexandrov LB Analysis of the genetic phylogeny of multifocal prostate cancer identifies multiple independent clonal expansions in neoplastic and morphologically normal prostate tissue. Nat Genet. (2015) 47:367–72. 10.1038/ng.3221 25730763 PMC4380509

[22] Pflueger D Terry S Sboner A Habegger L Esgueva R Lin PC. Discovery of non-ETS gene fusions in human prostate cancer using next-generation RNA sequencing. Genome Res. (2011) 21:56–67 10.1101/gr.110684.110 21036922 PMC3012926

[23] Taylor BS Schultz N Hieronymus H Gopalan A Xiao Y Carver BS. Integrative genomic profiling of human prostate cancer. Cancer Cell. (2010) 18:11–22. 10.1016/j.ccr.2010.05.026 20579941 PMC3198787

[24] Mahal BA Alshalalfa M Spratt DE Davicioni E Zhao SG Feng FY. Prostate cancer genomic-risk differences between African-American and white men across gleason scores. Eur Urol. (2019) 75:1038–40. 10.1016/j.eururo.2019.01.010 30683576

[25] Kim JS Gao X Rzhetsky A. RIDDLE: race and ethnicity imputation from disease history with deep learning. PLoS Comput Biol. (2018) 14:e1006106. 10.1371/journal.pcbi.1006106 29698408 PMC5940243

[26] Kingma DP Welling M Auto-encoding variational bayes. In: International Conference on Learning Representations. Banff, AB (2014).

[27] Box GE Tiao GC Bayesian Inference in Statistical Analysis. Hoboken, NJ: John Wiley & Sons (2011).

[28] Goodfellow I Pouget-Abadie J Mirza M Xu B Warde-Farley D Ozair S Generative adversarial nets. In: Ghahramani Z Welling M Cortes C Lawrence ND Weinberger KQ editors. Advances in Neural Information Processing Systems. Montreal, QC (2014). p. 2672–80.

[29] Lee C Mastronarde N van der Schaar M Estimation of individual treatment effect in latent confounder models via adversarial learning. In: Neural Information Processing Systems. Montreal, QC (2018).

[30] Louizos C Shalit U Mooij JM Sontag D Zemel R Welling Causal effect inference with deep latent-variable models. In: Guyon I Luxburg UV Bengio S Wallach H Fergus R Vishwanathan S Garnett R editors. Advances in Neural Information Processing Systems. Long Beach, CA (2017). p. 6446–6456.

[31] Yoon J Jordon J van der Schaar M GANITE: estimation of individualized treatment using generative adversarial nets. In: International Conference on Learning Representations. Vancouver, BC (2018).

[32] Kingma DP Ba J Adam: a method for stochastic optimization. arXiv [Preprint]. (2014) arXiv:1412.6980.

[33] Spratt DE Yousefi K Deheshi S Ross AE Den RB Schaeffer EM. Individual patient-level meta-analysis of the performance of the decipher genomic classifier in high-risk men after prostatectomy to predict development of metastatic disease. J Clin Oncol. (2017) 35:1991–8. 10.1200/JCO.2016.70.2811 28358655 PMC6530581

[34] National Cancer Institute (2019). Available online at: https://www.cancer.gov/news-events/cancer-currents-blog/2019/prostate-cancer-death-disparities-black-men

[35] Mahal BA Aizer AA Ziehr DR Hyatt AS Choueiri TK Hu JC. Racial disparities in prostate cancer–specific mortality in men with low-risk prostate cancer. Clin Genitourin Cancer. (2014) 12:e189–5. 10.1016/j.clgc.2014.04.003 24861952

[36] Tsivian M Banez LL Keto CJ Abern MR Qi P Gerber L. African-American men with low-grade prostate cancer have higher tumor burdens: results from the duke prostate center. Prostate Cancer Prostatic Dis. (2013) 16:91–4. 10.1038/pcan.2012.39 23032361

[37] Khani F Mosquera JM Park K Blattner M O'Reilly C MacDonald TY. Evidence for molecular differences in prostate cancer between African American and Caucasian men. Clin Cancer Res. (2014) 20:4925–34. 10.1158/1078-0432.CCR-13-2265 25056375 PMC4167562

[38] Sedarsky J Degon M Srivastava S Dobi A. Ethnicity and ERG frequency in prostate cancer. Nat Rev Urol. (2018) 15:125–31. 10.1038/nrurol.2017.140 28872154

[39] Powell IJ Dyson G Land S Ruterbusch J Bock CH Lenk S. Genes associated with prostate cancer are differentially expressed in African American and European American men. Cancer Epidemiol Biomarkers Prev. (2013) 22:891–7. 10.1158/1055-9965.EPI-12-1238 23515145 PMC4097306

[40] Ecke TH Schlechte HH Schiemenz K Sachs MD Lenk SV Rudolph BD. TP53 gene mutations in prostate cancer progression. Anticancer Res. (2010) 30:1579–86. 20592345

[41] Sirohi D Devine P Grenert JP van Ziffle J Simko JP Stohr BA. TP53 structural variants in metastatic prostatic carcinoma. PLoS ONE. (2019) 14:e0218618. 10.1371/journal.pone.0218618 31216325 PMC6583940

[42] Aggarwal CC Li Y Wang J Wang J Frequent pattern mining with uncertain data. In: Proceedings of the 15th ACM SIGKDD International Conference on Knowledge Discovery and Data Mining. Paris (2009). p. 29–38.

[43] Legler T Lehner W Schaffner J Krüger J Robust and distributed top-n frequent-pattern mining with SAP BW accelerator. Proceedings VLDB Endowment. (2009) 2:1438–49. 10.14778/1687553.1687571

