# Supplementary material for: Inferring Personalized and Race-Specific Causal Effects of Genomic Aberrations on Gleason Scores: A Deep Latent Variable Model
Source: Front Oncol. 2020 Mar 13;10:272. doi: 10.3389/fonc.2020.00272 (PMC7082760; doi:10.3389/fonc.2020.00272)
Supplement: Supplementary file 1 [file Data_Sheet_1.pdf]

Supplementary Materials for

"Inferring Personalized and Race-specific Causal Effects of Genomic Aberrations on Gleason Scores: A Deep Latent Variable Model"

Zhong Chen[1], Andrea Edwards[1], Chindo Hicks[2], Kun Zhang[1,3*]

[1]Department of Computer Science, Xavier University of Louisiana, New Orleans, LA, United States

[2]Department of Genetics, LSUHSC School of Medicine, New Orleans, LA, United States

[3]Bioinformatics Core of Xavier RCMI Center for Cancer Research, Xavier University of Louisiana, New Orleans, LA, United States

***Correspondence:** kzhang@xula.edu

Content:

- Supplementary Text S1: Sources of data and features for model training.
- Supplementary Text S2: Details of DLVM modeling, inference, and optimization.
- Supplementary Table S1: The comparison of race-specific RMSEs (± standard deviations) on the primary data: DLVM versus CEVAE.
- Supplementary Table S2: Genomic-aberration specific AICEs (± standard deviations) and p-values obtained via the paired t-test for EAs and AAs in the primary data for three grades of GS.
- Supplementary Table S3: Genomic-aberration specific Genomic-Risk Scores (GRSs) (± standard deviations) and p-values obtained via the paired t-test for EAs and AAs in the primary data for three grades of GS.
- Supplementary Table S4: Comparison of AICE and GRS patterns over all studied genomic aberrations on the primary data.
- Supplementary Table S5: Genomic-aberration specific AICEs (± standard deviations) and p-values obtained via the paired t-test for EAs and AAs in the validation data for three grades of GS.
- Supplementary Table S6: Comparison of AICE patterns on the primary and validation data over all studied genomic aberrations.
- Supplementary Figure S1: Boxplots of seven genomic-aberration specific AICEs of AAs and EAs in the primary data for different grades of GS.
- Supplementary Figure S2: Boxplots of seven genomic-aberration specific genomic-risk scores (GRSs) of AAs and EAs in the primary data for different grades of GS.
- Supplementary Figure S3: Boxplots of seven genomic-aberration specific AICEs of AAs and EAs in the validation data for different grades of GS.

Supplementary Text S1: Sources of data and features for model training.

As shown by Figure 2, DLVM is trained by two sets of variables – binary and continuous. The 46 binary variables are all about the mutation profiles. We obtain the data of those features from the supplementary files of (1), i.e. Table S1A.Annotation of “1-s2.0-S0092867415013392-mmc2.xls”. They are: ERG_status, ETV1_status, ETV4_status, FLI1_status, SPOP_mut, FOXA1_mut, MED12_mut, IDH1_mut, KMT2A_mut, KMT2C_mut, KMT2D_mut, KDM6A_mut, SETD2_mut, CHD1_mut, TP53_mut, PTEN_mut, PIK3CA_mut, BRAF_mut, HRAS_mut, CTNNB1_mut, AKT1_mut, BRCA1_mut, BRCA2_mut, BRCA1_germline_mut, BRCA2_germline_mut, CDKN1B_mut, RB1_mut, ZMYM3_mut, ATM_MUT, CDK12_MUT, FANCC_MUT, FANCD2_MUT, SPINK1_high, PTEN_CNA, TP53_CNA, CHD1_CNA, BRCA1_CNA, BRCA2_CNA, CDKN1B_CNA, RB1_CNA, CDK12_CNA, FANCD2_CNA, FAM175A_CNA, FANCC_CNA, RAD51C_CNA, and SPOPL_CNA. The 50 continuous variables include all clinical features from the file of “data_clinical_sample.txt” and 31 gene expression values from the file of “data_RNA_Seq_v2_expression_median.txt” in “prad_tcga_pub” downloaded from CBioPortal. Those 31 genes are a subset of the aforementioned molecules with genomic aberations. These genes are: ERG, ETV1, ETV4, FLI1, SPOP, FOXA1, MED12, IDH1, KDM6A, SETD2, CHD1, TP53, PTEN, PIK3CA, BRAF, HRAS, CTNNB1, AKT1, BRCA1, BRCA2, CDKN1B, RB1, ZMYM3, ATM, CDK12, FANCC, FANCD2, SPINK1, FAM175A, RAD51C, and SPOPL.

Supplementary Text S2: Details of DLVM modeling, inference, and optimization.

The model illustrated in Figure 2 suggests the following factorization:

$$\begin{aligned} p(\boldsymbol{Z}, \boldsymbol{z}_1, \boldsymbol{z}_2, \boldsymbol{x}_1, \boldsymbol{x}_2, t, y) &= p(\boldsymbol{z}_1, \boldsymbol{z}_2)p(\boldsymbol{x}_1, \boldsymbol{x}_2|\boldsymbol{z}_1, \boldsymbol{z}_2)p(\boldsymbol{Z}|\boldsymbol{z}_1, \boldsymbol{z}_2)p(t|\boldsymbol{Z})p(y|t, \boldsymbol{Z}) \\ &= p(\boldsymbol{z}_1)p(\boldsymbol{z}_2)p(\boldsymbol{x}_1|\boldsymbol{z}_1)p(\boldsymbol{x}_2|\boldsymbol{z}_2)p(\boldsymbol{Z}|\boldsymbol{z}_1, \boldsymbol{z}_2)p(t|\boldsymbol{Z})p(y|t, \boldsymbol{Z}) \end{aligned} \quad (1)$$

where $p(\boldsymbol{z}_1)$ is a discrete prior distribution (i.e., Bernoulli distribution), and $p(\boldsymbol{z}_2)$ is a continuous prior distribution (i.e., Gaussian distribution). As Figure 2 suggests that, the observed variables $\boldsymbol{x}_1$ (or $\boldsymbol{x}_2$) are conditionally independent given the latent variables $\boldsymbol{z}_1$ (or $\boldsymbol{z}_2$). Accordingly, the conditional distributions $p(\boldsymbol{x}_1|\boldsymbol{z}_1)$ and $p(\boldsymbol{x}_2|\boldsymbol{z}_2)$ are the Bernoulli and Gaussian distributions, respectively.

Without loss of generality, $\boldsymbol{x}_1$ and $\boldsymbol{x}_2$ are generated by some random processes in two steps. First, the unobserved latent variables $\boldsymbol{z}_1$ and $\boldsymbol{z}_2$ are drawn from the prior distribution $p(\boldsymbol{z}_1, \boldsymbol{z}_2)$; and then $\boldsymbol{x}_1$ and $\boldsymbol{x}_2$ are sampled from the conditional distribution $p(\boldsymbol{x}_1, \boldsymbol{x}_2|\boldsymbol{z}_1, \boldsymbol{z}_2)$. The joint probability distribution is $p(\boldsymbol{x}_1, \boldsymbol{x}_2, \boldsymbol{z}_1, \boldsymbol{z}_2) = p(\boldsymbol{x}_1, \boldsymbol{x}_2|\boldsymbol{z}_1, \boldsymbol{z}_2)p(\boldsymbol{z}_1, \boldsymbol{z}_2)$, where the density functions $p(\boldsymbol{x}_1, \boldsymbol{x}_2|\boldsymbol{z}_1, \boldsymbol{z}_2)$ and $p(\boldsymbol{z}_1, \boldsymbol{z}_2)$ are differential almost everywhere w.r.t. both $\boldsymbol{x}_1$, $\boldsymbol{x}_2$ and $\boldsymbol{z}_1$, $\boldsymbol{z}_2$. Using maximum likelihood estimation, we are able to approximate the corresponding parameters. However, it is quite common that the marginalization of $\boldsymbol{z}_1$ or $\boldsymbol{z}_2$ is intractable, particularly in cases of a moderately complicated likelihood function $p(\boldsymbol{x}_1, \boldsymbol{x}_2|\boldsymbol{z}_1, \boldsymbol{z}_2)$ (e.g., a neural network). A popular strategy dealing with such intractability is the variational Bayesian method, i.e., to derive a tractable lower bound to approximate the intractable marginal likelihood, so as to optimize the variational lower bound (i.e., the Evidence Lower Bound (ELBO)) as follows.

$$\begin{aligned} ELBO &= \mathcal{L}(\boldsymbol{x}_1, \boldsymbol{x}_2, t, y) \\ &= \mathbb{E}_{(\boldsymbol{Z}, \boldsymbol{z}_1, \boldsymbol{z}_2) \sim q(\boldsymbol{Z}, \boldsymbol{z}_1, \boldsymbol{z}_2|\boldsymbol{x}_1, \boldsymbol{x}_2, t, y)}[\log p(\boldsymbol{Z}|\boldsymbol{z}_1, \boldsymbol{z}_2) + \log p(\boldsymbol{z}_1, \boldsymbol{z}_2) \\ &+ \log p(\boldsymbol{x}_1, \boldsymbol{x}_2|\boldsymbol{z}_1, \boldsymbol{z}_2) + \log p(t|\boldsymbol{Z}) + \log p(y|t, \boldsymbol{Z}) \\ &- \log q(\boldsymbol{Z}, \boldsymbol{z}_1, \boldsymbol{z}_2|\boldsymbol{x}_1, \boldsymbol{x}_2, t, y)] \leq \log p(\boldsymbol{x}_1, \boldsymbol{x}_2, t, y) \end{aligned} \quad (2)$$

where $q(\boldsymbol{Z}, \boldsymbol{z}_1, \boldsymbol{z}_2|\boldsymbol{x}_1, \boldsymbol{x}_2, t, y)$ is the introduced distribution function that attempts to approximate $p(\boldsymbol{Z}, \boldsymbol{z}_1, \boldsymbol{z}_2|\boldsymbol{x}_1, \boldsymbol{x}_2, t, y)$. Since the observed data and the variational distribution parameters $q(\boldsymbol{Z}, \boldsymbol{z}_1, \boldsymbol{z}_2, \boldsymbol{x}_1, \boldsymbol{x}_2, t, y)$ are obtained through various neural network functions, the ELBO is the objective function to maximize w.r.t. those functions. This will require the use of back-propagation (2) combined with a gradient-based optimizer (e.g., Adagrad, RMSprop or ADAM (3)). For the optimization, the distributions $p(\boldsymbol{z}_1)$ and $p(\boldsymbol{z}_2)$ correspond to the latent hidden confounders, $p(t|\boldsymbol{Z})$ is described as $p(t|\boldsymbol{Z}) = Bern\left(softmax\left(net(\boldsymbol{Z})\right)\right)$. For a continuous outcome (such as PSA), we parameterize the probability distribution as a Gaussian distribution with the mean and variance, i.e., $p(y|t, \boldsymbol{Z}) = \mathcal{N}(\mu = \hat{\mu}, \sigma = \hat{\sigma})$. For a discrete outcome (such as GS), we use a Bernoulli distribution to parameterize the probability distribution, i.e., $p(y|t, \boldsymbol{Z}) = Bern(\pi = \hat{\pi})$. Using this method, we can infer the unknown outcome given an untruthful/hypothetical intervention (via the deep encoder) and approximate the true outcome given a truthful/factual intervention (by the deep decoder).

In the following, we mathematically prove Eq. (2) by deriving the ELBO. Using the factorization (1) and Bayesian rule, we have:

$$p(\boldsymbol{Z}, \boldsymbol{z}_1, \boldsymbol{z}_2|\boldsymbol{x}_1, \boldsymbol{x}_2, t, y) = \frac{p(\boldsymbol{Z}, \boldsymbol{z}_1, \boldsymbol{z}_2, \boldsymbol{x}_1, \boldsymbol{x}_2, t, y)}{p(\boldsymbol{x}_1, \boldsymbol{x}_2, t, y)} \quad (3)$$

We introduce a discriminative model $q(\boldsymbol{Z}, \boldsymbol{z}_1, \boldsymbol{z}_2|\boldsymbol{x}_1, \boldsymbol{x}_2, t, y)$ to approximate $p(\boldsymbol{Z}, \boldsymbol{z}_1, \boldsymbol{z}_2|\boldsymbol{x}_1, \boldsymbol{x}_2, t, y)$. Thus, our objective function is:

$$\min KL(q(\boldsymbol{Z}, \boldsymbol{z}_1, \boldsymbol{z}_2|\boldsymbol{x}_1, \boldsymbol{x}_2, t, y) \| p(\boldsymbol{Z}, \boldsymbol{z}_1, \boldsymbol{z}_2|\boldsymbol{x}_1, \boldsymbol{x}_2, t, y)) \quad (4)$$

That is,

$KL(q(\boldsymbol{Z}, \boldsymbol{z}_1, \boldsymbol{z}_2|\boldsymbol{x}_1, \boldsymbol{x}_2, t, y) \| p(\boldsymbol{Z}, \boldsymbol{z}_1, \boldsymbol{z}_2|\boldsymbol{x}_1, \boldsymbol{x}_2, t, y)) = \iiint q(\boldsymbol{Z}, \boldsymbol{z}_1, \boldsymbol{z}_2|\boldsymbol{x}_1, \boldsymbol{x}_2, t, y) \log \frac{q(\boldsymbol{Z}, \boldsymbol{z}_1, \boldsymbol{z}_2|\boldsymbol{x}_1, \boldsymbol{x}_2, t, y)}{p(\boldsymbol{Z}, \boldsymbol{z}_1, \boldsymbol{z}_2|\boldsymbol{x}_1, \boldsymbol{x}_2, t, y)} d\boldsymbol{z}_1 d\boldsymbol{z}_2 d\boldsymbol{Z}$, then, we have

$KL(q(\boldsymbol{Z}, \boldsymbol{z}_1, \boldsymbol{z}_2|\boldsymbol{x}_1, \boldsymbol{x}_2, t, y) \| p(\boldsymbol{Z}, \boldsymbol{z}_1, \boldsymbol{z}_2|\boldsymbol{x}_1, \boldsymbol{x}_2, t, y)) = \iiint q(\boldsymbol{Z}, \boldsymbol{z}_1, \boldsymbol{z}_2|\boldsymbol{x}_1, \boldsymbol{x}_2, t, y)[\log q(\boldsymbol{Z}, \boldsymbol{z}_1, \boldsymbol{z}_2|\boldsymbol{x}_1, \boldsymbol{x}_2, t, y) - \log p(\boldsymbol{Z}, \boldsymbol{z}_1, \boldsymbol{z}_2, \boldsymbol{x}_1, \boldsymbol{x}_2, t, y) + \log p(\boldsymbol{x}_1, \boldsymbol{x}_2, t, y)] d\boldsymbol{z}_1 d\boldsymbol{z}_2 d\boldsymbol{Z} = \iiint q(\boldsymbol{Z}, \boldsymbol{z}_1, \boldsymbol{z}_2|\boldsymbol{x}_1, \boldsymbol{x}_2, t, y) \log \frac{q(\boldsymbol{Z}, \boldsymbol{z}_1, \boldsymbol{z}_2|\boldsymbol{x}_1, \boldsymbol{x}_2, t, y)}{p(\boldsymbol{Z}, \boldsymbol{z}_1, \boldsymbol{z}_2, \boldsymbol{x}_1, \boldsymbol{x}_2, t, y)} d\boldsymbol{z}_1 d\boldsymbol{z}_2 d\boldsymbol{Z} + \log p(\boldsymbol{x}_1, \boldsymbol{x}_2, t, y) = -\mathbb{E}_{(\boldsymbol{Z}, \boldsymbol{z}_1, \boldsymbol{z}_2) \sim q(\boldsymbol{Z}, \boldsymbol{z}_1, \boldsymbol{z}_2|\boldsymbol{x}_1, \boldsymbol{x}_2, t, y)} \log \frac{p(\boldsymbol{Z}, \boldsymbol{z}_1, \boldsymbol{z}_2, \boldsymbol{x}_1, \boldsymbol{x}_2, t, y)}{q(\boldsymbol{Z}, \boldsymbol{z}_1, \boldsymbol{z}_2|\boldsymbol{x}_1, \boldsymbol{x}_2, t, y)} + \log p(\boldsymbol{x}_1, \boldsymbol{x}_2, t, y) \geq 0$,

Hence,

$ELBO = \mathcal{L}(\boldsymbol{x}_1, \boldsymbol{x}_2, t, y) = \mathbb{E}_{(\boldsymbol{Z}, \boldsymbol{z}_1, \boldsymbol{z}_2) \sim q(\boldsymbol{Z}, \boldsymbol{z}_1, \boldsymbol{z}_2|\boldsymbol{x}_1, \boldsymbol{x}_2, t, y)} \log \frac{p(\boldsymbol{Z}, \boldsymbol{z}_1, \boldsymbol{z}_2, \boldsymbol{x}_1, \boldsymbol{x}_2, t, y)}{q(\boldsymbol{Z}, \boldsymbol{z}_1, \boldsymbol{z}_2|\boldsymbol{x}_1, \boldsymbol{x}_2, t, y)} = \log p(\boldsymbol{x}_1, \boldsymbol{x}_2, t, y) - KL(q(\boldsymbol{Z}, \boldsymbol{z}_1, \boldsymbol{z}_2|\boldsymbol{x}_1, \boldsymbol{x}_2, t, y) \| p(\boldsymbol{Z}, \boldsymbol{z}_1, \boldsymbol{z}_2|\boldsymbol{x}_1, \boldsymbol{x}_2, t, y)) \leq \log p(\boldsymbol{x}_1, \boldsymbol{x}_2, t, y)$,

Therefore, $\mathcal{L}(\boldsymbol{x}_1, \boldsymbol{x}_2, t, y)$ is a lower bound of $\log p(\boldsymbol{x}_1, \boldsymbol{x}_2, t, y)$, called the variational lower bound. When $\mathcal{L}(\boldsymbol{x}_1, \boldsymbol{x}_2, t, y) \to \log p(\boldsymbol{x}_1, \boldsymbol{x}_2, t, y)$, we have $KL(q(\boldsymbol{Z}, \boldsymbol{z}_1, \boldsymbol{z}_2|\boldsymbol{x}_1, \boldsymbol{x}_2, t, y) \| p(\boldsymbol{Z}, \boldsymbol{z}_1, \boldsymbol{z}_2|\boldsymbol{x}_1, \boldsymbol{x}_2, t, y)) \to 0$, and $q(\boldsymbol{Z}, \boldsymbol{z}_1, \boldsymbol{z}_2|\boldsymbol{x}_1, \boldsymbol{x}_2, t, y) \to p(\boldsymbol{Z}, \boldsymbol{z}_1, \boldsymbol{z}_2|\boldsymbol{x}_1, \boldsymbol{x}_2, t, y)$.

Thus, we can derive the ELBO based on Eq. (1):
$ELBO = \mathbb{E}_{(\boldsymbol{Z}, \boldsymbol{z}_1, \boldsymbol{z}_2) \sim q(\boldsymbol{Z}, \boldsymbol{z}_1, \boldsymbol{z}_2|\boldsymbol{x}_1, \boldsymbol{x}_2, t, y)} \log \frac{p(\boldsymbol{Z}, \boldsymbol{z}_1, \boldsymbol{z}_2, \boldsymbol{x}_1, \boldsymbol{x}_2, t, y)}{q(\boldsymbol{Z}, \boldsymbol{z}_1, \boldsymbol{z}_2|\boldsymbol{x}_1, \boldsymbol{x}_2, t, y)} = \mathbb{E}_{(\boldsymbol{Z}, \boldsymbol{z}_1, \boldsymbol{z}_2) \sim q(\boldsymbol{Z}, \boldsymbol{z}_1, \boldsymbol{z}_2|\boldsymbol{x}_1, \boldsymbol{x}_2, t, y)}[\log p(\boldsymbol{z}_1, \boldsymbol{z}_2) + \log p(\boldsymbol{x}_1, \boldsymbol{x}_2|\boldsymbol{z}_1, \boldsymbol{z}_2) + \log p(\boldsymbol{Z}|\boldsymbol{z}_1, \boldsymbol{z}_2) + \log p(t|\boldsymbol{Z}) + \log p(y|t, \boldsymbol{Z}) - \log q(\boldsymbol{Z}, \boldsymbol{z}_1, \boldsymbol{z}_2|\boldsymbol{x}_1, \boldsymbol{x}_2, t, y)]$, this proves Eq. (2).

Supplementary Table S1: The comparison of race-specific RMSEs (± standard deviations) on the primary data: DLVM versus CEVAE.

Methods		DLVM		CEVAE	
**Genomic Aberrations**	**Gene**	**EA**	**AA**	**EA**	**AA**
**Gene Fusions**	**ERG**	2.39±0.08	1.94±0.06	3.28±0.20	2.12±0.12
**Somatic Mutations**	**SPOP**	2.59±0.11	1.75±0.11	3.27±0.22	2.07±0.10
	**TP53**	2.80±0.13	1.94±0.15	3.43±0.21	2.19±0.16
	**FOXA1**	2.70±0.06	1.75±0.12	3.11±0.29	2.03±0.16
	**ATM**	2.41±0.17	1.62±0.14	3.14±0.20	2.55±0.10
	**BRCA2**	2.91±0.14	1.75±0.08	3.68±0.22	2.45±0.16
	**PTEN**	2.79±0.10	2.13±0.08	3.38±0.12	2.68±0.12
**Germline Mutations**	**BRCA2**	2.51±0.18	1.94±0.09	3.89±0.17	2.61±0.15
	**BRCA1**	2.40±0.10	2.13±0.12	3.07±0.12	2.19±0.11
**CNAs**	**LCP1**	2.40±0.15	1.63±0.14	3.39±0.21	2.13±0.16
	**ERG**	2.80±0.13	1.81±0.12	3.37±0.20	2.23±0.21
	**PTEN**	2.70±0.17	1.73±0.15	3.44±0.22	2.41±0.23
	**FOXA1**	2.38±0.10	2.01±0.12	3.54±0.21	2.34±0.23

Supplementary Table S2: Genomic-aberration specific AICEs (± standard deviations) and p-values obtained via the paired t-test for EAs and AAs in the primary data for three grades of GS.

Genomic Aberrations	Genes	GS Grades	EAs	AAs	p values	CI
**Gene Fusions**	**ERG**	**GS<=6**	1.59±0.21	1.86±0.26	p=0.009	[-0.527,-0.080]
		**GS=7**	2.18±0.14	1.92±0.19	p=0.007	[0.066,0.382]
		**GS>=8**	2.97±0.25	1.99±0.20	p<0.001	[0.801,1.230]
**Somatic Mutations**	**SPOP**	**GS<=6**	1.53±0.08	1.76±0.14	p<0.001	[-0.377,-0.162]
		**GS=7**	2.05±0.12	1.63±0.10	p<0.001	[0.335,0.544]
		**GS>=8**	2.75±0.15	1.82±0.18	p<0.001	[0.756,1.070]
	**TP53**	**GS<=6**	2.02±0.18	2.11±0.24	p=0.199	[-0.258,0.056]
		**GS=7**	2.26±0.12	1.86±0.10	p<0.001	[0.313,0.476]
		**GS>=8**	3.00±0.25	2.47±0.28	p<0.001	[0.333,0.725]
	**FOXA1**	**GS<=6**	1.22±0.08	1.30±0.14	p=0.005	[-0.152,-0.029]
		**GS=7**	1.36±0.12	1.42±0.10	p=0.078	[-0.112,0.006]
		**GS>=8**	1.41±0.15	1.86±0.08	p<0.001	[-0.509,-0.379]
	**ATM**	**GS<=6**	1.87±0.18	1.62±0.24	p=0.002	[0.097,0.378]
		**GS=7**	2.10±0.12	1.74±0.10	p<0.001	[0.286,0.432]
		**GS>=8**	2.83±0.25	1.98±0.28	p<0.001	[0.673,1.025]
	**BRCA2**	**GS<=6**	1.63±0.08	1.77±0.12	p<0.001	[-0.210,-0.077]
		**GS=7**	1.83±0.11	1.86±0.13	p=0.301	[-0.120,0.038]
		**GS>=8**	2.02±0.15	2.54±0.11	p<0.001	[-0.605,-0.433]
	**PTEN**	**GS<=6**	1.15±0.08	1.18±0.04	p=0.115	[-0.055,0.006]

		**GS=7**	1.46±0.08	1.20±0.10	p<0.001	[0.215,0.303]
		**GS>=8**	1.60±0.15	1.32±0.08	p<0.001	[0.240,0.358]
**Germline Mutations**	**BRCA2**	**GS<=6**	1.15±0.08	1.36±0.04	p<0.001	[-0.261,-0.159]
		**GS=7**	1.66±0.12	1.07±0.10	p<0.001	[0.499,0.678]
		**GS>=8**	1.99±0.15	2.38±0.18	p<0.001	[-0.517,-0.250]
	**BRCA1**	**GS<=6**	0.95±0.09	1.03±0.04	p<0.001	[-0.112,-0.038]
		**GS=7**	0.95±0.08	0.93±0.10	p=0.581	[-0.035,0.062]
		**GS>=8**	1.24±0.12	1.29±0.11	p=0.114	[-0.110,0.012]
**CNAs**	**LCP1**	**GS<=6**	1.50±0.21	1.66±0.26	p=0.060	[-0.285,0.006]
		**GS=7**	2.37±0.14	1.86±0.19	p<0.001	[0.419,0.625]
		**GS>=8**	2.81±0.25	1.45±0.20	p<0.001	[1.200,1.479]
	**ERG**	**GS<=6**	1.60±0.11	1.78±0.12	p<0.001	[-0.236,-0.127]
		**GS=7**	2.13±0.10	1.95±0.09	p<0.001	[0.131,0.221]
		**GS>=8**	2.93±0.15	2.46±0.10	p<0.001	[0.417,0.537]
	**PTEN**	**GS<=6**	1.69±0.21	1.64±0.22	p=0.443	[-0.074,0.165]
		**GS=7**	2.98±0.20	1.82±0.19	p<0.001	[1.057,1.273]
		**GS>=8**	2.18±0.25	2.10±0.20	p=0.275	[-0.057,0.194]
	**FOXA1**	**GS<=6**	1.68±0.11	1.87±0.16	p<0.001	[-0.272,-0.104]
		**GS=7**	2.10±0.14	2.05±0.19	p=0.387	[-0.058,0.147]
		**GS>=8**	2.08±0.15	2.41±0.16	p<0.001	[-0.424,-0.234]

Supplementary Table S3: Genomic-aberration specific Genomic-Risk Scores (GRSs) (± standard deviations) and p-values obtained via the paired t-test for EAs and AAs in the primary data for three grades of GS.

Genomic Aberrations	Genes	GS Grades	EAs	AAs	p values	CI
Gene Fusions	ERG	GS<=6	0.23±0.03	0.30±0.06	p<0.001	[-0.091,-0.033]
		GS=7	0.38±0.04	0.34±0.05	p=0.004	[0.015,0.071]
		GS>=8	0.51±0.10	0.44±0.08	p=0.024	[0.009,0.120]
Somatic Mutations	SPOP	GS<=6	0.26±0.03	0.33±0.04	p<0.001	[-0.090,-0.049]
		GS=7	0.40±0.05	0.36±0.03	p=0.002	[0.016,0.063]
		GS>=8	0.55±0.08	0.43±0.06	p<0.001	[0.079,0.160]
	TP53	GS<=6	0.30±0.04	0.32±0.04	p=0.191	[-0.050,0.010]
		GS=7	0.42±0.03	0.36±0.05	p<0.001	[0.027,0.090]
		GS>=8	0.48±0.07	0.43±0.06	p=0.046	[0.001,0.100]
	FOXA1	GS<=6	0.24±0.04	0.27±0.03	p=0.002	[-0.046,-0.011]
		GS=7	0.32±0.03	0.35±0.04	p<0.001	[-0.049,-0.014]
		GS>=8	0.44±0.04	0.50±0.06	p<0.001	[-0.088,-0.038]
	ATM	GS<=6	0.26±0.04	0.20±0.03	p<0.001	[0.045,0.080]
		GS=7	0.33±0.03	0.28±0.04	p<0.001	[0.029,0.065]
		GS>=8	0.40±0.06	0.35±0.05	p<0.001	[0.024,0.080]
	BRCA2	GS<=6	0.25±0.02	0.29±0.03	p<0.001	[-0.065,-0.029]
		GS=7	0.32±0.03	0.33±0.04	p=0.173	[0.042,0.008]
		GS>=8	0.35±0.02	0.44±0.05	p<0.001	[-0.139,-0.085]
	PTEN	GS<=6	0.25±0.04	0.24±0.03	p=0.410	[-0.018,0.043]

		GS=7	0.38±0.03	0.33±0.04	p=0.003	[0.017,0.078]
		GS>=8	0.45±0.06	0.38±0.05	p=0.004	[0.025,0.120]
Germline Mutations	BRCA2	GS<=6	0.20±0.02	0.25±0.03	p<0.001	[-0.064,-0.034]
		GS=7	0.33±0.03	0.30±0.04	p=0.006	[0.010,0.052]
		GS>=8	0.39±0.06	0.42±0.05	p=0.068	[-0.064,0.002]
	BRCA1	GS<=6	0.19±0.04	0.23±0.02	p<0.001	[-0.053,-0.022]
		GS=7	0.31±0.03	0.32±0.04	p=0.195	[-0.028,0.006]
		GS>=8	0.43±0.04	0.44±0.05	p=0.309	[-0.033,0.011]
CNAs	LCP1	GS<=6	0.28±0.04	0.29±0.05	p=0.464	[-0.038,0.018]
		GS=7	0.38±0.06	0.33±0.04	p=0.003	[0.019,0.082]
		GS>=8	0.56±0.07	0.48±0.06	p<0.001	[0.039,0.121]
	ERG	GS<=6	0.24±0.03	0.29±0.04	p<0.001	[-0.073,-0.022]
		GS=7	0.36±0.04	0.33±0.05	p=0.049	[0.002,0.065]
		GS>=8	0.54±0.08	0.45±0.07	p=0.002	[0.033,0.141]
	PTEN	GS<=6	0.32±0.04	0.31±0.03	p=0.150	[-0.005,0.033]
		GS=7	0.41±0.06	0.36±0.04	p<0.001	[0.030,0.085]
		GS>=8	0.54±0.04	0.52±0.05	p=0.179	[-0.008,0.040]
	FOXA1	GS<=6	0.22±0.02	0.28±0.03	p<0.001	[-0.094,-0.031]
		GS=7	0.35±0.03	0.34±0.04	p=0.423	[-0.012,0.030]
		GS>=8	0.44±0.04	0.50±0.06	p<0.001	[-0.077,-0.046]

Supplementary Table S4: Comparison of AICE and GRS patterns over all studied genomic aberrations on the primary data. Differences in two genes are highlighted in circles.
√ denotes that AICE or GRS of AAs is statistically significantly higher than that of EAs.
× denotes that AICE or GRS of EAs is statistically significantly higher than that of AAs.
– denotes that there is no statistically significant difference in AICE or GRS between AAs and EAs.

Metrics	Aberrations	Genes	GS<=6	GS=7	GS>=8
AICEs	Fusions	ERG	√	×	×
	Somatic Mutations	SPOP	√	×	×
		TP53	–	×	×
		FOXA1	√	–	√
		ATM	×	×	×
		BRCA2	√	–	√
		PTEN	–	×	×
	Germline Mutations	BRCA2	√	×	√
		BRCA1	√	–	–
	CNAs	LCP1	–	×	×
		ERG	√	×	×
		PTEN	–	×	–
		FOXA1	√	–	√
Genomic-risk Scores (GRSs)	Fusions	ERG	√	×	×
	Somatic Mutations	SPOP	√	×	×
		TP53	–	×	×
		FOXA1	√	√	√
		ATM	×	×	×
		BRCA2	√	–	√
		PTEN	–	×	×
	Germline Mutations	BRCA2	√	×	–
		BRCA1	√	–	–
	CNAs	LCP1	–	×	×
		ERG	√	×	×
		PTEN	–	×	–
		FOXA1	√	–	√

Supplementary Table S5: Genomic-aberration specific AICEs (± standard deviations) and p-values obtained via the paired t-test for 414 EAs and 65 AAs in the validation data for three grades of GS.

Genomic Aberrations	Genes	GS Grades	EAs	AAs	p values	CI
Gene Fusions	ERG	GS<=6	2.36±0.18	2.48±0.15	p=0.013	[-0.219,-0.028]
		GS=7	2.65±0.25	2.51±0.23	p=0.024	[-0.029,0.306]
		GS>=8	2.96±0.35	2.71±0.26	p=0.009	[0.063,0.418]
Somatic Mutations	SPOP	GS<=6	2.06±0.26	2.15±0.18	p=0.042	[-0.224,0.011]
		GS=7	2.26±0.18	2.24±0.20	p=0.656	[-0.072,0.112]
		GS>=8	2.74±0.28	2.55±0.24	p=0.004	[0.063,0.0315]
	TP53	GS<=6	2.24±0.30	2.26±0.22	p=0.772	[-0.184,0.138]
		GS=7	2.60±0.34	2.26±0.24	p<0.001	[0.152,0.508]
		GS>=8	2.83±0.32	2.44±0.30	p<0.001	[0.200,0.576]
	FOXA1	GS<=6	1.82±0.06	1.90±0.08	p<0.001	[-0.116,-0.041]
		GS=7	2.08±0.08	2.10±0.10	p=0.371	[-0.063,0.024]
		GS>=8	2.03±0.12	2.54±0.28	p<0.001	[-0.612,-0.382]
	ATM	GS<=6	2.38±0.14	2.02±0.11	p<0.001	[0.283,0.422]
		GS=7	2.46±0.16	2.15±0.12	p<0.001	[0.221,0.378]
		GS>=8	2.63±0.30	2.30±0.22	p<0.001	[0.164,0.456]
	BRCA2	GS<=6	1.73±0.08	1.94±0.09	p<0.001	[-0.278,-0.144]
		GS=7	1.86±0.16	2.12±0.13	p<0.001	[-0.371,-0.142]
		GS>=8	2.04±0.21	2.71±0.36	p<0.001	[-0.918,-0.455]
	PTEN	GS<=6	1.70±0.06	1.68±0.05	p=0.387	[-0.024,0.061]

		**GS=7**	1.95±0.08	1.60±0.05	p<0.001	[0.294,0.396]
		**GS>=8**	2.15±0.20	1.83±0.16	p<0.001	[0.174,0.453]
**Germline Mutations**	**BRCA2**	**GS<=6**	1.63±0.04	1.82±0.06	p<0.001	[-0.225,-0.170]
		**GS=7**	1.71±0.05	1.53±0.05	p<0.001	[0.158,0.202]
		**GS>=8**	1.76±0.15	2.65±0.32	p<0.001	[-1.086,-0.819]
	**BRCA1**	**GS<=6**	1.43±0.02	1.78±0.05	p<0.001	[-0.365,-0.328]
		**GS=7**	1.62±0.06	1.61±0.05	p=0.374	[-0.016,0.043]
		**GS>=8**	1.90±0.20	1.91±0.18	p=0.788	[-0.080,0.104]
**CNAs**	**LCP1**	**GS<=6**	2.43±0.24	2.40±0.22	p=0.692	[-0.129,0.192]
		**GS=7**	2.65±0.31	2.45±0.26	p=0.045	[0.005,0.403]
		**GS>=8**	2.81±0.40	2.57±0.28	p=0.042	[0.010,0.490]
	**ERG**	**GS<=6**	2.13±0.16	2.35±0.12	p<0.001	[-0.302,-0.128]
		**GS=7**	2.60±0.30	2.40±0.21	p=0.031	[0.016,0.319]
		**GS>=8**	2.90±0.28	2.70±0.26	p=0.018	[0.036,0.369]
	**PTEN**	**GS<=6**	2.33±0.33	2.31±0.32	p=0.793	[-0.138,0.180]
		**GS=7**	2.56±0.21	2.40±0.22	p=0.020	[0.026,0.293]
		**GS>=8**	2.67±0.34	2.74±0.38	p=0.408	[-0.249,0.103]
	**FOXA1**	**GS<=6**	2.42±0.25	2.56±0.26	p=0.027	[-0.270,-0.017]
		**GS=7**	2.52±0.30	2.50±0.28	p=0.708	[-0.117,0.171]
		**GS>=8**	2.83±0.36	2.61±0.30	p=0.005	[0.076,0.405]

Supplementary Table S6: Comparison of AICE patterns on the primary and validation data over all studied genomic aberrations. Differences in two genes are highlighted in circles.
√ denotes that AICE or GRS of AAs is statistically significantly higher than that of EAs.
× denotes that AICE or GRS of EAs is statistically significantly higher than that of AAs.
– denotes that there is no statistically significant difference in AICE or GRS between AAs and EAs.

Metrics	Aberrations	Genes	GS<=6	GS=7	GS>=8
AICEs (270 AA, 43 AA)	Fusions	ERG	√	×	×
	Somatic Mutations	SPOP	√	×	×
		TP53	–	×	×
		FOXA1	√	–	√
		ATM	×	×	×
		BRCA2	√	–	√
		PTEN	–	×	×
	Germline Mutations	BRCA2	√	×	√
		BRCA1	√	–	–
	CNAs	LCP1	–	×	×
		ERG	√	×	×
		PTEN	–	×	–
		FOXA1	√	–	√
AICEs (144 EA, 22 AA)	Fusions	ERG	√	×	×
	Somatic Mutations	SPOP	√	–	×
		TP53	–	×	×
		FOXA1	√	–	√
		ATM	×	×	×
		BRCA2	√	√	√
		PTEN	–	×	×
	Germline Mutations	BRCA2	√	×	√
		BRCA1	√	–	–
	CNAs	LCP1	–	×	×
		ERG	√	×	×
		PTEN	–	×	–
		FOXA1	√	–	×

Supplementary Figure S1: **Boxplots of seven genomic-aberration specific AICEs of AAs and EAs in the primary data for different grades of GS.** The paired t-test with the significance level $\alpha = 0.05$ is utilized for hypothesis testing, where the null hypothesis is "H=0: a genomic-aberration specific AICEs are not differentiated over racial groups", and the alternative hypothesis is "H=1: a genomic-aberration specific AICEs are differentiated over racial groups". CI: Confidence Interval.

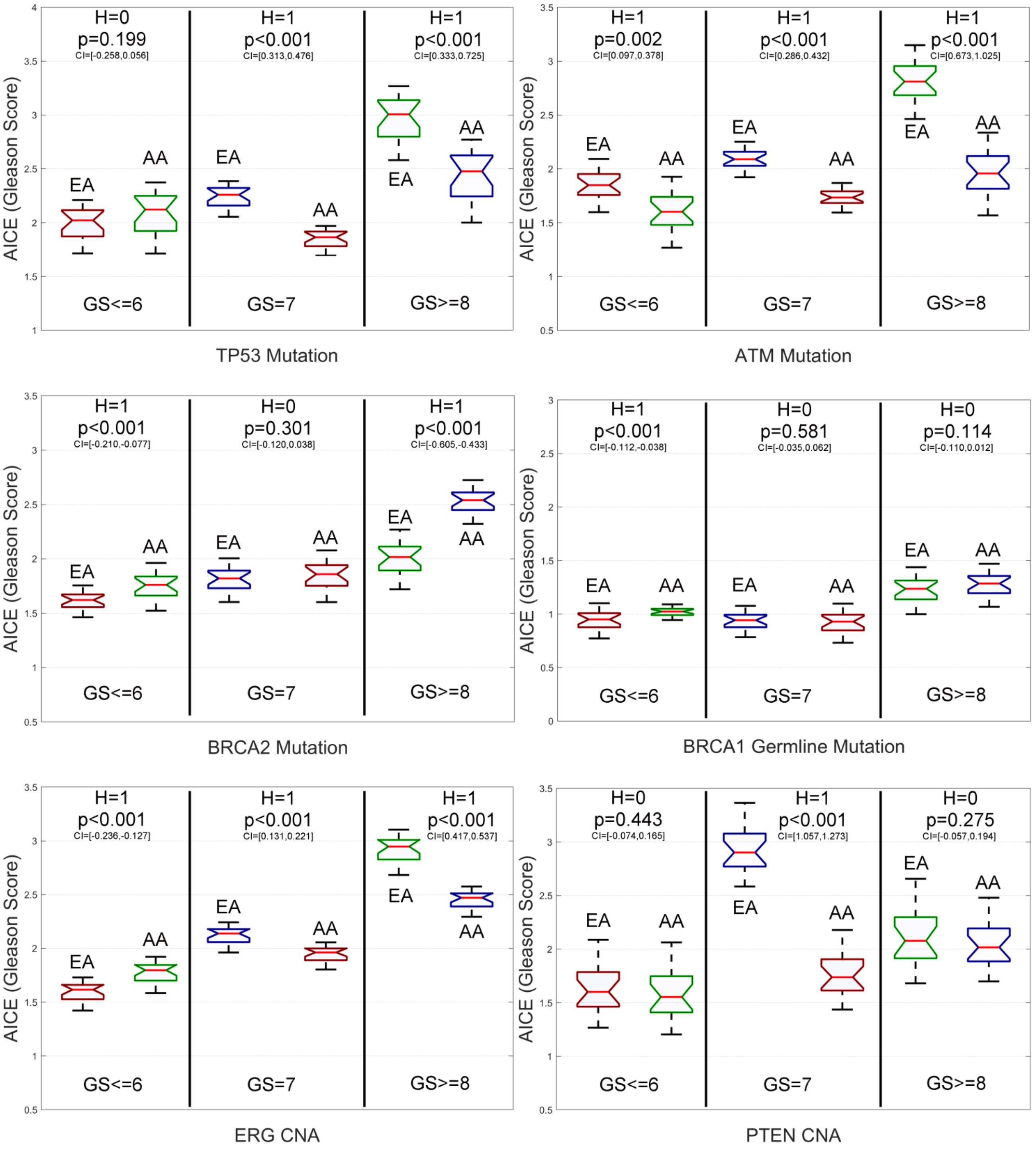

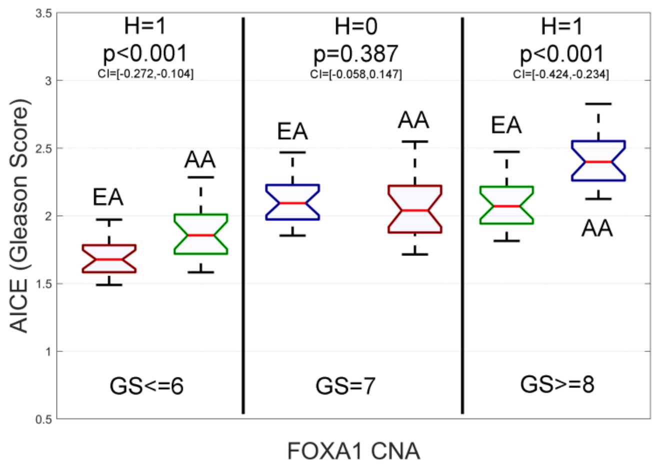
H=1
p<0.001
CI=[-0.272,-0.104]
H=0
p=0.387
CI=[-0.058,0.147]
H=1
p<0.001
CI=[-0.424,-0.234]
EA
AA
EA
AA
EA
AA
GS<=6
GS=7
GS>=8
AICE (Gleason Score)
FOXA1 CNA

Supplementary Figure S2: Boxplots of seven genomic-aberration specific genomic-risk scores (GRSs) of AAs and EAs in the primary data for different grades of GS. The paired t-test with the significance level $\alpha = 0.05$ is utilized for hypothesis testing, where the null hypothesis is “H=0: a genomic-aberration specific GRSs are not differentiated over racial groups”, and the alternative hypothesis is “H=1: a genomic-aberration specific GRSs are differentiated over racial groups”. CI: Confidence Interval.

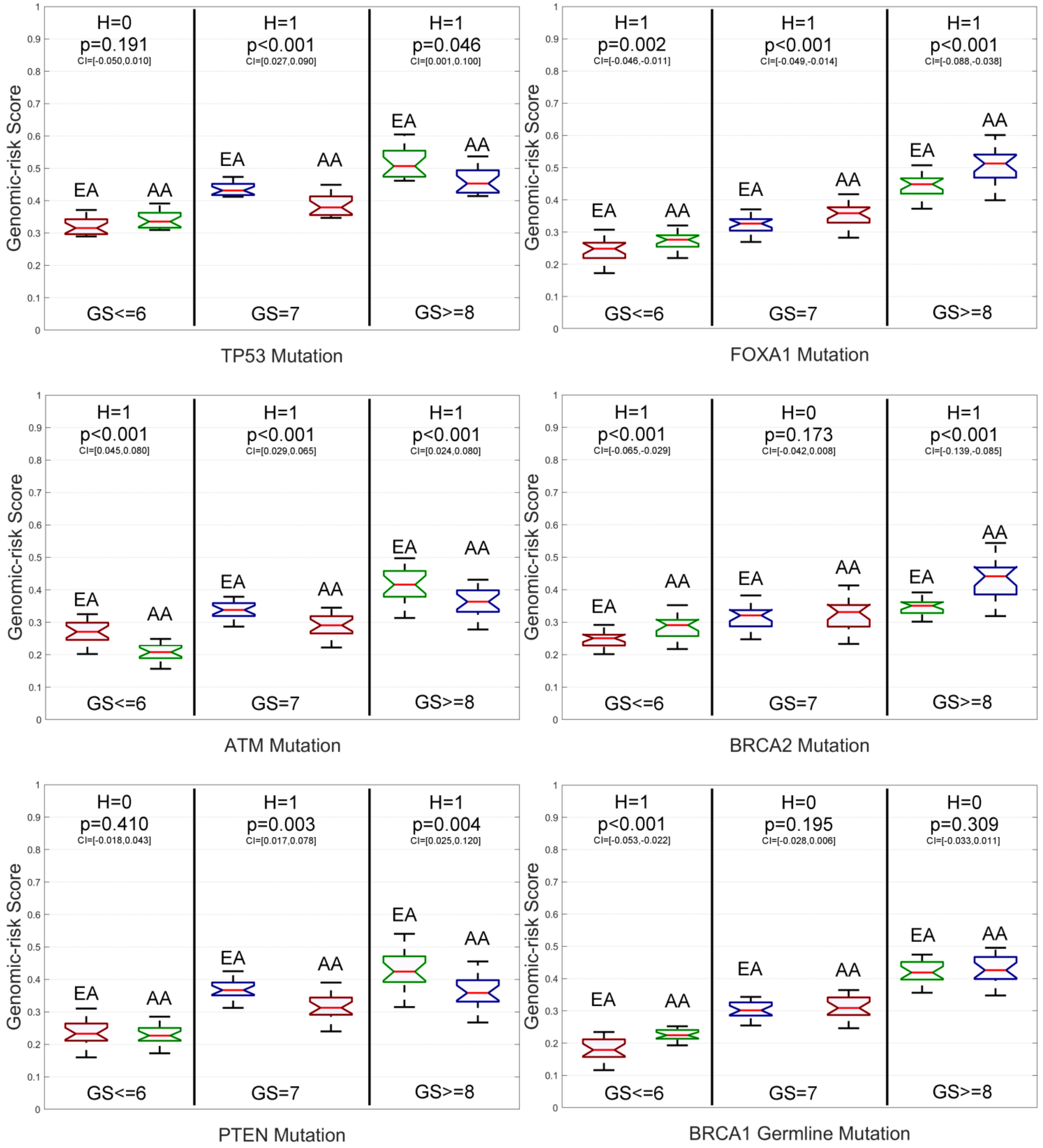

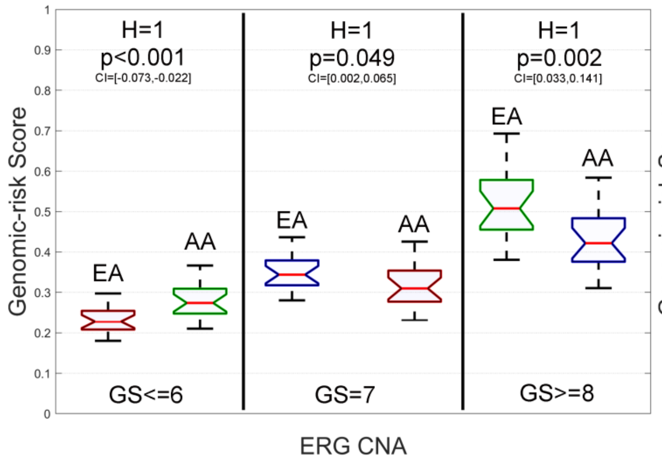
Genomic-risk Score
H=1
p<0.001
CI=[-0.073,-0.022]
H=1
p=0.049
CI=[0.002,0.065]
H=1
p=0.002
CI=[0.033,0.141]
EA
AA
GS<=6
GS=7
GS>=8
ERG CNA

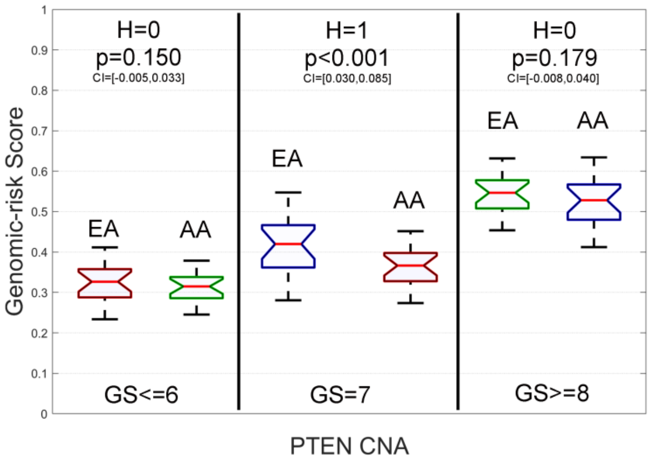
Genomic-risk Score
H=0
p=0.150
CI=[-0.005,0.033]
H=1
p<0.001
CI=[0.030,0.085]
H=0
p=0.179
CI=[-0.008,0.040]
EA
AA
GS<=6
GS=7
GS>=8
PTEN CNA

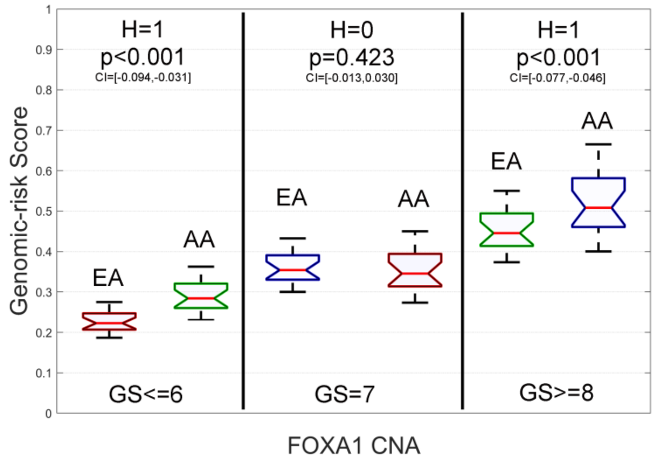
Genomic-risk Score
H=1
p<0.001
CI=[-0.094,-0.031]
H=0
p=0.423
CI=[-0.013,0.030]
H=1
p<0.001
CI=[-0.077,-0.046]
EA
AA
GS<=6
GS=7
GS>=8
FOXA1 CNA

Supplementary Figure S3: **Boxplots of seven genomic-aberration specific AICEs of AAs and EAs in the validation data for different grades of GS.** The paired t-test with the significance level $\alpha = 0.05$ is utilized for hypothesis testing, where the null hypothesis is “H=0: a genomic-aberration specific AICEs are not differentiated over racial groups”, and the alternative hypothesis is “H=1: a genomic-aberration specific AICEs are differentiated over racial groups”. CI: Confidence Interval.

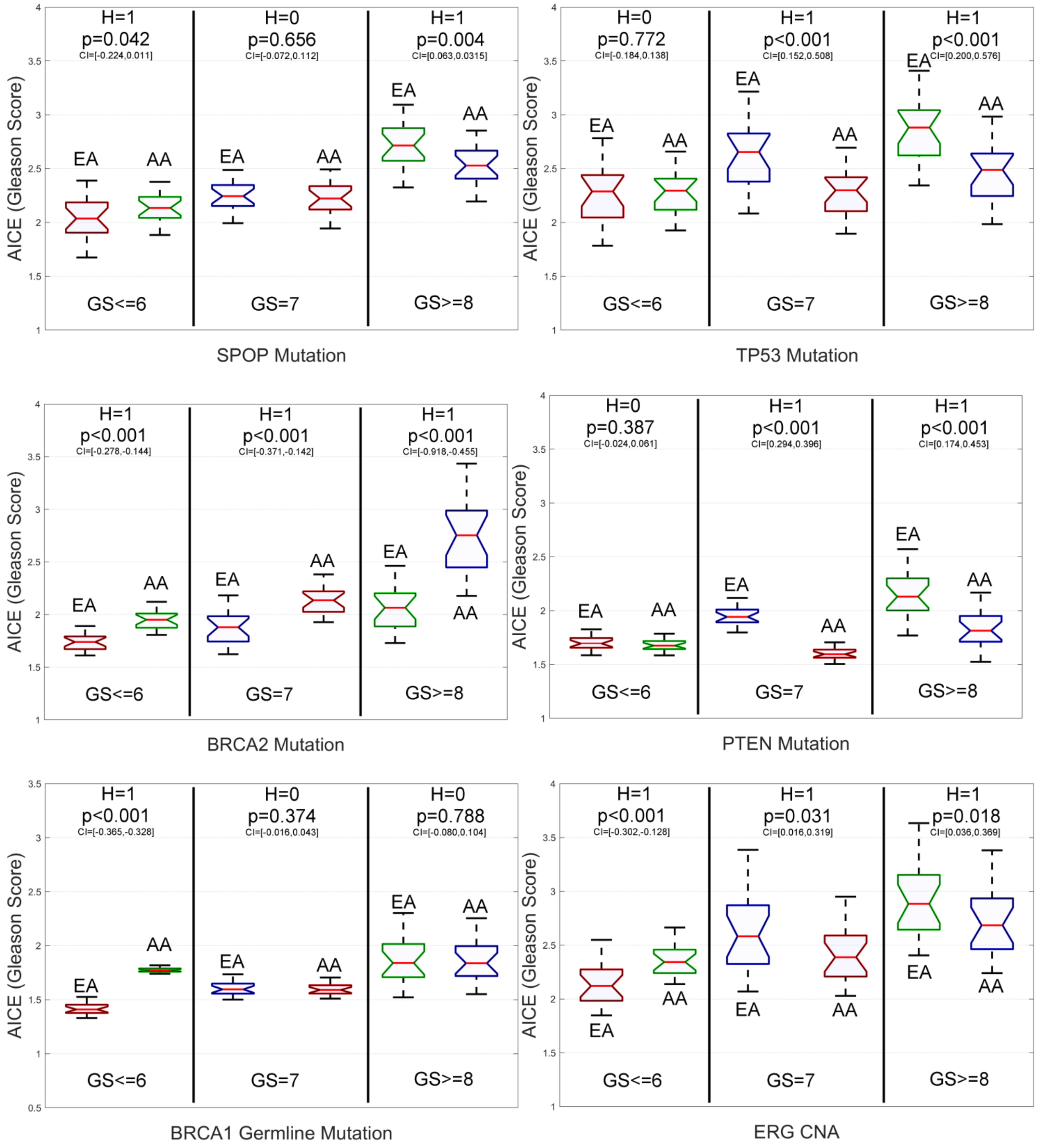

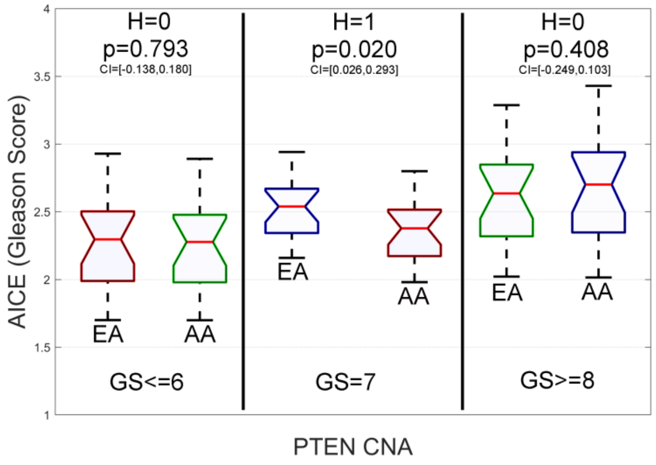

References

1. Abeshouse A, Ahn J, Akbani R, Ally A, Amin S, Andry CD, Annala M, Aprikian A, Armenia J, Arora A, Auman JT. The molecular taxonomy of primary prostate cancer. *Cell* (2015) 163:1011–1025. doi: 10.1016/j.cell.2015.10.025
2. Hinton G, Osindero S, Welling M, Teh YW. Unsupervised discovery of nonlinear structure using contrastive backpropagation. *Cogn. Sci.* (2006) 30: 725–731. doi: 10.1207/s15516709cog0000_76
3. Ruder S. An overview of gradient descent optimization algorithms. *arXiv preprint* (2016) arXiv:1609.04747.
